# Coherent helicity-dependent spin-phonon oscillations in the ferromagnetic van der Waals crystal CrI_3_

**DOI:** 10.1038/s41467-022-31786-3

**Published:** 2022-08-02

**Authors:** P. Padmanabhan, F. L. Buessen, R. Tutchton, K. W. C. Kwock, S. Gilinsky, M. C. Lee, M. A. McGuire, S. R. Singamaneni, D. A. Yarotski, A. Paramekanti, J.-X. Zhu, R. P. Prasankumar

**Affiliations:** 1 Center for Integrated Nanotechnologies, Los Alamos National Laboratory, Los Alamos, NM USA; 2 Department of Physics, University of Toronto, Toronto, Ontario Canada; 3 The Fu Foundation School of Engineering and Applied Science, Columbia University, New York, NY USA; 4 Materials Science and Technology Division, Oak Ridge National Laboratory, Oak Ridge, TN USA; 5 Department of Physics, The University of Texas at El Paso, El Paso, TX USA; 6 Deep Science Fund, Intellectual Ventures, Bellevue, WA USA

**Keywords:** Magnetic properties and materials, Ferromagnetism, Magnetic properties and materials, Magneto-optics, Near-infrared spectroscopy

## Abstract

The discovery of two-dimensional systems hosting intrinsic magnetic order represents a seminal addition to the rich landscape of van der Waals materials. CrI_3_ is an archetypal example, where the interdependence of structure and magnetism, along with strong light-matter interactions, provides a new platform to explore the optical control of magnetic and vibrational degrees of freedom at the nanoscale. However, the nature of magneto-structural coupling on its intrinsic ultrafast timescale remains a crucial open question. Here, we probe magnetic and vibrational dynamics in bulk CrI_3_ using ultrafast optical spectroscopy, revealing spin-flip scattering-driven demagnetization and strong transient exchange-mediated interactions between lattice vibrations and spin oscillations. The latter yields a coherent spin-coupled phonon mode that is highly sensitive to the driving pulse’s helicity in the magnetically ordered phase. Our results elucidate the nature of ultrafast spin-lattice coupling in CrI_3_ and highlight its potential for applications requiring high-speed control of magnetism at the nanoscale.

## Abstract (web summary)

CrI3 is a van der Waals material which exhibits magnetic ordering down to the monolayer limit. Here, using ultrafast optical spectroscopy, Padmanabhan and Buessen et al. investigate the coupling between the magnetically ordered spins and lattice distortions, finding a coherent spin-coupled phonon mode.

## Introduction

Since its first demonstration^1^, the ultrafast manipulation of magnetism has been a major topic of research, with significant efforts aimed at unraveling the nature of dynamic demagnetization^2,3^, the excitation of collective magnetic modes^4,5^, and the realization of all-optical switching of magnetic phases^6–8^. The recent emergence of two-dimensional (2D) van der Waals (vdW) materials hosting intrinsic long-range magnetic order^9–11^ presents a new, relatively unexplored platform for investigating these phenomena in systems where the interplay between structural order and exchange interactions plays a pivotal role. Moreover, the inherently strong light-matter interactions typical of vdW materials open exciting new possibilities for exploring the fundamental properties of magneto-structural coupling at the nanoscale under the influence of intense optical excitation^12–14^. This in turn has the potential to yield revolutionary leaps in low-dimensional magneto-optical devices for future storage and quantum information applications, especially when used in heterostructured device architectures^15^.

**Fig. 1:**
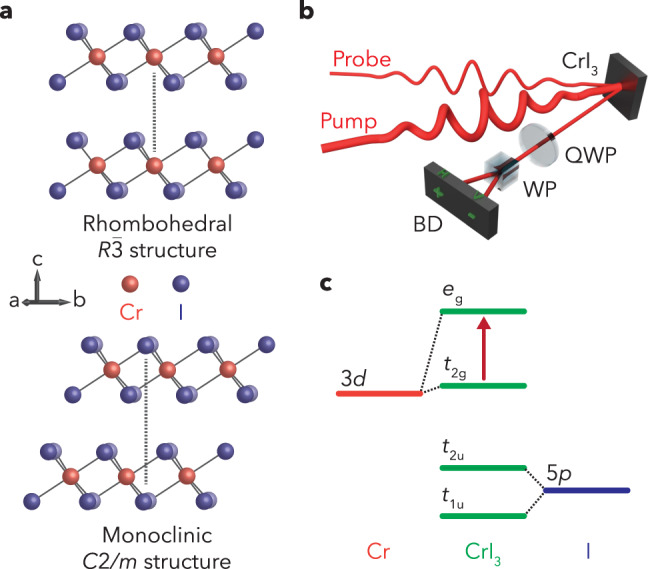
CrI_3_ structural and electronic properties and TRPR experimental scheme. Schematics of **a** the crystal structure of the rhombohedral ($R\bar{3}$) and monoclinic ($C2/m$) phases of CrI_3_, **b** the femtosecond TRPR experiment, where QWP is a quarter wave plate, WP is a Wollaston prism, and BD is a balanced detector, and **c** the relevant energy levels in CrI_3_, where the red arrow denotes optical transitions at $\sim 1.5\,{{{{\rm{eV}}}}}$ that generate excited electrons and holes. Dashed lines indicate the dominant atomic orbital character of the ligand-field split levels. Transitions between the ${e}_{{{{{{\rm{g}}}}}}}$, ${t}_{1{{{{{\rm{u}}}}}}}$, and ${t}_{2{{{{{\rm{u}}}}}}}$ levels significantly contribute to coherent phonon generation.

CrI_3_ is a prototypical example of a magnetic vdW material hosting out-of-plane ferromagnetic (FM) order down to the monolayer limit^10^ stabilized by uniaxial anisotropy, which opens a gap in the low energy zone-center magnon spectrum^16^. Even more intriguing is the fact that the magnetic phase is highly dependent on the number of atomic layers. This is due to the nature of the fundamental Cr-Cr superexchange interactions^17^, which stabilize an antiferromagnetic (AFM) phase with broken inversion symmetry in even-layered crystals. This gives rise to unique nonlinear optical phenomena^18^ and highlights the strong interdependence between the various crystal structures (Fig. 1a), magnetic orders, and optical responses present in CrI_3_. Additionally, the presence of phonon-induced lattice distortions that can modulate the length scale of spin-spin interactions may unlock the possibility for dynamic light-driven coherent coupling between the lattice and spin degrees of freedom^19^.

Here, we uncover the nature of this coupling by measuring the transient magnetization and coherent vibrational dynamics in CrI_3_ after femtosecond optical photoexcitation. We find that the demagnetization dynamics are driven by spin-flip scattering, while coherent pump helicity-dependent oscillations in the time-resolved polarization rotation (TRPR) signal highlight the strong influence of magnetic order on the *c*-axis ${A}_{1{{{{{\rm{g}}}}}}}$ optical phonon mode. This can be interpreted within the framework of a dynamic spin-lattice coupling mechanism, which opens a unique pathway for manipulating magnetic order through the vibrational degree of freedom, with significant implications for future nanoscale opto-magnetic applications.

## Results

### Ultrafast Demagnetization

**Fig. 2:**
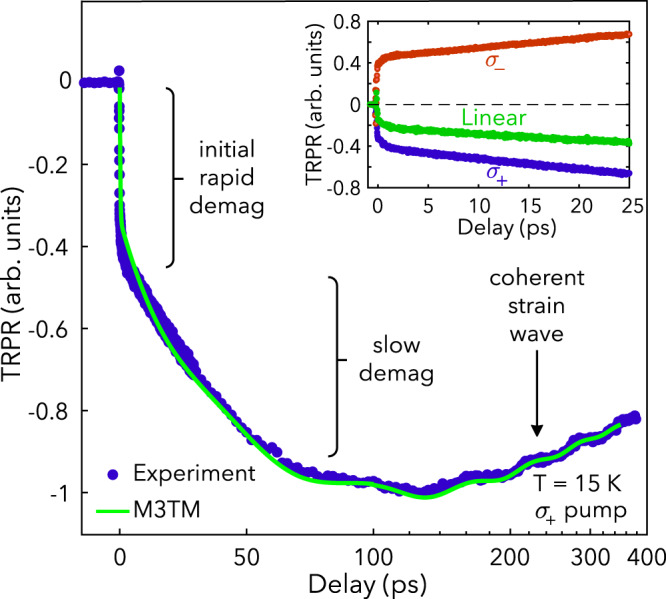
Ultrafast photoinduced demagnetization in CrI_3_. Time-resolved polarization rotation signal under ${\sigma }_{+}$ pumping at $T=15\,{{{{\rm{K}}}}}$, where the blue dots are experimental data and the green trace is a fit using the M3TM (see the SI for more details). The oscillatory component in the M3TM fit curve was obtained by separately fitting the oscillatory component of the experimental data with a decaying sinusoidal function. The inset shows the magnetization dynamics on shorter timescales under ${\sigma }_{+}$ (blue), ${\sigma }_{-}$ (red), and linearly polarized (green) pumping at $T=15\,{{{{\rm{K}}}}}$.

In our experiments, $1.55\,{{{{\rm{eV}}}}}$ ($800\,{{{{\rm{nm}}}}}$), $85\,{{{{\rm{fs}}}}}$ pump and probe pulses (measured at the sample position) were focused at near-normal incidence onto as-grown bulk-like flakes of CrI_3_ (Curie temperature, ${T}_{c}=61\,{{{{\rm{K}}}}}$) (Fig. 1b). Figure 2 shows the resulting TRPR signal obtained in the FM phase at $T=15\,{{{{\rm{K}}}}}$ under right circularly polarized (${\sigma }_{+}$) pumping. We observe a rapid sub-picosecond decrease in the magnetization, a further reduction over tens of picoseconds, a $16\,{{{{\rm{GHz}}}}}$ oscillation due to a coherent strain wave, and an eventual nanosecond timescale recovery. This two-step demagnetization process, referred to as type-II dynamics, has been observed in other ferromagnets^20,21^ and occurs when demagnetization is not completed before electron-phonon equilibration^2^.

More insight can be obtained by considering the optical transitions in CrI_3_ associated with $1.55\,{{{{\rm{eV}}}}}$ photoexcitation. The absorption spectrum of bulk CrI_3_ shows a weak resonance peak at $1.5\,{{{{\rm{eV}}}}}$^22^, attributed to transitions between the partially filled ${t}_{2{{{{{\rm{g}}}}}}}$ and unfilled ${e}_{{{{{{\rm{g}}}}}}}$ levels resulting from the octahedral ligand field-induced splitting of the Cr^3+^ *d*-orbital, as shown in Fig. 1c. Despite the even parity of these states, this transition is allowed due to mixing with various odd-parity states^22^, and may also be associated with a low-lying bright charge-transfer exciton^23^. Regardless, below ${T}_{c}$ the $1.55\,{{{{\rm{eV}}}}}$ pump pulse drives transitions predominantly involving majority-spin states, due to significant spin polarization of the valence and conduction bands^23,24^. This leads to a strong preferential absorption of ${\sigma }_{+}$ light in spin-up domains at $1.55\,{{{{\rm{eV}}}}}$^23^.

The helicity-dependent absorptivity of CrI_3_ suggests that the ultrafast magnetic response should be nearly negligible for left circularly polarized (${\sigma }_{-}$) pump pulses. However, as seen in the inset of Fig. 2, we observe that the TRPR signal under ${\sigma }_{-}$ pumping has a sign opposite to the ${\sigma }_{+}$ case. This helicity-dependent response is preserved for all temperatures $T\, < \,{T}_{c}$. We attribute this to the presence of multiple domains within the photoexcited region^15,25^; i.e., spin-down domains preferentially absorb ${\sigma }_{-}$ photons, leading to their subsequent demagnetization. Nevertheless, horizontally polarized pumping yields a negative signal (Fig. 2 inset), implying that the volume fraction of spin-up domains is higher in the probed region.

Accordingly, upon absorption of a ${\sigma }_{+}$ pulse, majority-spin electrons and holes are excited in CrI_3_. Due to spin-orbit coupling (SOC), the wave functions of these carriers are a mixture of pure spin states^26^. This allows for a finite probability of Elliott-Yafet spin-flip scattering processes^27^, which mediate spin relaxation, particularly in the hole population due to the comparatively small valence band exchange splitting^23^. Furthermore, the photoexcited carrier density of ${\sim}{10}^{19}\,{{{{\rm{cm}}}}}^{-3}$ exceeds the Mott density criterion, given the nanometer-scale exciton radius^23^, leading to a transient quasi-metallic state. This makes it possible to describe ultrafast dynamics in CrI_3_ using the microscopic three-temperature model (M3TM), in which the excited electronic system supplies the energy for demagnetization, while interactions with the lattice allow for angular momentum dissipation^2^. A fit to the demagnetization dynamics with the M3TM, shown in Fig. 2, accurately reproduces both demagnetization steps with a spin-flip probability of ${a}_{{{{{{\rm{sf}}}}}}}=0{{{{\rm{.}}}}}175$, consistent with other materials demonstrating type-II dynamics^20,21^. More detail is included in the supplementary information (SI).

### Coherent dynamics

**Fig. 3:**
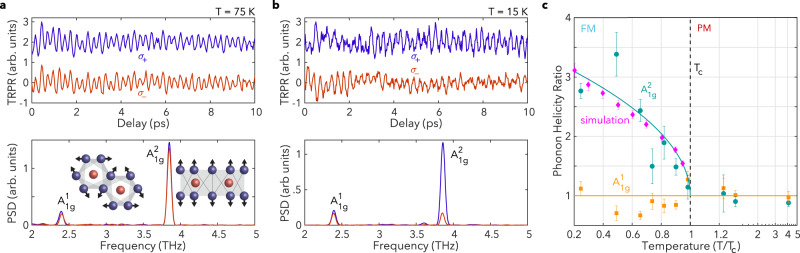
Coherent spin-coupled phonon dynamics as a function of temperature. Oscillations in the time domain signals (upper panel) after subtraction of the demagnetization background, under ${\sigma }_{+}$ (blue) and ${\sigma }_{-}$ (red) pumping, and their corresponding power spectral densities (PSDs, lower panel) at **a** $T=75\,{{{{\rm{K}}}}}$ and **b** $T=15\,{{{{\rm{K}}}}}$. The inset of **a** shows a schematic of the eigenvectors associated with the two phonon modes. **c** The ${\sigma }_{+}/{\sigma }_{-}$ ratio of the integrated Fourier transform peaks of the measured signal at the ${A}_{1{{{{{\rm{g}}}}}}}^{1}$ (orange) and ${A}_{1{{{{{\rm{g}}}}}}}^{2}$ (green) mode frequencies, and the simulated helicity-dependent ratio at the ${A}_{1{{{{{\rm{g}}}}}}}^{2}$ frequency (pink) vs. normalized temperature. The solid green line is a fit using a FM order-parameter-like function $\propto \sqrt{{T}_{c}-T}$ and the solid orange line is a guide to the eye. Error bars for the experimental data in **c** were obtained from a bootstrap sampling analysis.

We now turn our attention to the coherent dynamics. The top panel of Fig. 3a shows the TRPR signal at $T=75\,{{{{\rm{K}}}}}\ > \ {T}_{c}$ under ${\sigma }_{+}$ and ${\sigma }_{-}$ pumping after subtracting the non-oscillatory background. Above ${T}_{c}$, the background signal is relatively small (as shown in Supplementary Fig. 4), and most likely originates from optical effects such as pump-induced birefringence, while below ${T}_{c}$, the background is large and primarily due to the ultrafast demagnetization phenomena discussed in the previous section. We observe pronounced coherent oscillations in the time domain, and the corresponding power spectral densities (PSDs) plotted in the bottom panel reveal two distinct modes at ~$2.37\,{{{{\rm{THz}}}}}$ and ~$3.87\,{{{{\rm{THz}}}}}$, corresponding to in-plane (${A}_{1{{{{{\rm{g}}}}}}}^{1}$) and *c*-axis (${A}_{1{{{{{\rm{g}}}}}}}^{2}$) Raman-active phonons, respectively^28–31^. These modes are excited via impulsive stimulated Raman scattering (ISRS), which is permitted by their ${A}_{1{{{{{\rm{g}}}}}}}$ symmetry^30,31^.

Notably, the PSD of both modes is nearly identical for both pump helicities above ${T}_{c}$. However, as shown in Fig. 3b, this symmetry is broken below ${T}_{c}$, where ${\sigma }_{-}$ pumping leads to a significantly smaller ${A}_{1{{{{{\rm{g}}}}}}}^{2}$ amplitude. In contrast, the amplitude of the ${A}_{1{{{{{\rm{g}}}}}}}^{1}$ mode remains relatively invariant to the choice of pump polarization. The temperature and pump-helicity dependent asymmetry in the phonon amplitude can be clearly seen in Fig. 3c, where we plot the ${\sigma }_{+}/{\sigma }_{-}$ ratio of the integrated spectral peaks of both oscillatory modes vs. *T*. Here, the ${A}_{1{{{{{\rm{g}}}}}}}^{2}$ ratio below ${T}_{c}$ consistently follows a FM order-parameter-like function, $\propto \sqrt{{T}_{c}-T}$. This highlights the strong sensitivity of the coherent amplitude of the ${A}_{1{{{{{\rm{g}}}}}}}^{2}$ mode to the underlying magnetic order in the system. In contrast, the ${A}_{1{{{{{\rm{g}}}}}}}^{1}$ mode ratio shows minimal variation with temperature. This difference is striking and suggests that the helicity dependence of the ${A}_{1{{{{{\rm{g}}}}}}}^{2}$ mode does not originate from a dominant optical effect (i.e., the larger absorptivity of ${\sigma }_{+}$ light in CrI_3_ below ${T}_{c}$), as this would impact the two modes in a similar manner. Additionally, we note that time-reversal symmetry breaking associated with magnetic ordering can lead to non-zero off-diagonal tensor elements for the ${A}_{1{{{{{\rm{g}}}}}}}$ modes^32^, which in turn can lead to helicity-dependent asymmetries in the equilibrium spectral response^31,33^. However, this is unlikely to be the origin of the pump helicity-dependent effects we observe in our experiments. First, the influence of off-diagonal tensor elements should be present for all modes of ${A}_{1{{{{{\rm{g}}}}}}}$ symmetry, in stark contrast to our results where only the *c*-axis mode is sensitive to the pump polarization; this strongly implies that mode symmetry alone cannot account for our observations. Second, recent ab initio calculations show that the magnitude of the off-diagonal elements is highly sensitive to the photon energy, becoming non-trivial only in the vicinity of the dominant ligand-metal charge transfer resonances above $2\,{{{{\rm{eV}}}}}$^34^. Our photon energy is far below these resonances, further supporting an alternative mechanism for the helicity-dependent effects observed here. Finally, we note that fitting our time-domain data with a multi-component decaying sinusoidal model reveals the presence of an additional oscillatory mode below ${T}_{c}$, consistent with recent reports of AFM order dependent modes at $3.73\,{{{{\rm{THz}}}}}$, close to the ${A}_{1{{{{{\rm{g}}}}}}}^{2}$ mode frequency^31,33,35^. However, this mode does not show a pronounced helicity dependence in our measurements (more detail is provided in Supplementary Fig. 5 and the associated discussion).

To understand the clear sensitivity of the ${A}_{1{{{{{\rm{g}}}}}}}^{2}$ mode to the underlying magnetic order, we recall that this mode corresponds to a *c*-axis oscillation of iodine atoms (shown schematically in the lower panel of Fig. 3a) leading to an oscillatory trigonal distortion of the CrI_6_ octahedra. This distortion can transiently modulate spin exchange interactions through anisotropy- or exchange-mediated pathways^19,36^. We confirmed this using density functional theory (DFT) to calculate $\varDelta E={E}_{{{{{{\rm{FM}}}}}}}-{E}_{{{{{{\rm{AFM}}}}}}}$, the energy difference per spin between FM and AFM configurations, which is a measure of the overall exchange strength. Tracking its dependence on the average equilibrium lattice displacement, *X*, of the ${A}_{1{{{{{\rm{g}}}}}}}^{2}$ phonon yields $\partial \varDelta E/\partial X \approx -32\,{{{{\rm{meV/}}}}}\AA$ per Cr atom, signaling the importance of spin-lattice coupling in CrI_3_. Here, $X\, > \, 0$ denotes trigonal compression of the CrI_6_ octahedra.

To explain the helicity-dependent TRPR, we then developed a spin-phonon model for CrI_3_. This describes interacting $S=3/2$ Cr moments on a layered honeycomb lattice that are coupled to the local ${A}_{1{{{{{\rm{g}}}}}}}^{2}$ Einstein phonon displacements. The total Hamiltonian is $H={H}_{{{{{{\rm{ph}}}}}}}+{H}_{{{{{{\rm{sp}}}}}}}$, with the phonon term

1 $${H}_{{{{{{\rm{ph}}}}}}}=\mathop{\sum}\limits_{{{{{{\bf{r}}}}}}}\frac{{P}_{{{{{{\bf{r}}}}}}}^{2}}{2M}+\frac{M{\Omega }^{2}{X}_{{{{{{\bf{r}}}}}}}^{2}}{2}.$$

Here ${X}_{{{\bf{r}}}}$ and ${P}_{{{\bf{r}}}}$ are the displacement and momentum of the Einstein phonon at lattice site **r**, ${M} \approx 6.3 \times {10}^{-25}\,{{{{\rm{kg}}}}}$ is the mass of the three iodine atoms per Cr spin, and ${\Omega} \approx 3.87\,{{{{\rm{THz}}}}}$ is the ${A}_{1{{{{{\rm{g}}}}}}}^{2}$ phonon frequency. The spin Hamiltonian is ${H}_{{{{{{\rm{sp}}}}}}}={H}_{{{{{{\rm{ex}}}}}}}+{H}_{{{{{{\rm{si}}}}}}}$, with two-spin exchange

2 $${H}_{{{{{{\rm{ex}}}}}}}=	 \;\mathop{\sum}\limits _{{\left\langle {{{{{{\bf{rr}}}}}}}^{{\prime} }\right\rangle }_{\gamma }}\left[{\tilde{J}}_{H1,{{{{{\bf{r}}}}}}{{{{{{\bf{r}}}}}}}^{{{{\prime} }}}}{{{{{{\bf{S}}}}}}}_{{{{{{\bf{r}}}}}}}\cdot {{{{{{\bf{S}}}}}}}_{{{{{{{\bf{r}}}}}}}^{{{{\prime} }}}}+{J}_{K}\,{S}_{{{{{{\bf{r}}}}}}}^{\gamma }\,{S}_{{{{{{{\bf{r}}}}}}}^{{\prime} }}^{\gamma }\right]+\mathop{\sum}\limits_{\left\langle \left\langle {{{{{{\bf{rr}}}}}}}^{{\prime} }\right\rangle \right\rangle }{J}_{H2}{{{{{{\bf{S}}}}}}}_{{{{{{\bf{r}}}}}}}\cdot {{{{{{\bf{S}}}}}}}_{{{{{{{\bf{r}}}}}}}^{{{{\prime}}}}}\\ 	+\mathop{\sum}\limits _{\left\langle \left\langle {{{{{{\bf{rr}}}}}}}^{{\prime} }\right\rangle \right\rangle }{J}_{D}{\hat{{{{{{\bf{D}}}}}}}}_{{{{{{\bf{r}}}}}}{{{{{{\bf{r}}}}}}}^{{\prime} }}\cdot {{{{{{\bf{S}}}}}}}_{{{{{{\bf{r}}}}}}}\times {{{{{{\bf{S}}}}}}}_{{{{{{{\bf{r}}}}}}}^{{\prime} }}+\mathop{\sum}\limits_{\left\langle \left\langle \left\langle {{{{{{\bf{rr}}}}}}}^{{\prime} }\right\rangle \right\rangle \right\rangle }{J}_{H3}{{{{{{\bf{S}}}}}}}_{{{{{{\bf{r}}}}}}}\cdot {{{{{{\bf{S}}}}}}}_{{{{{{{\bf{r}}}}}}}^{{{{\prime} }}}}+\mathop{\sum}\limits_{{\left\langle {{{{{{\bf{rr}}}}}}}^{{\prime} }\right\rangle }_{c}}{J}_{{Hc}}{{{{{{\bf{S}}}}}}}_{{{{{{\bf{r}}}}}}}\cdot {{{{{{\bf{S}}}}}}}_{{{{{{{\bf{r}}}}}}}^{{{{\prime} }}}},$$

where ${\left\langle {{{{{{\bf{rr}}}}}}}^{{\prime} }\right\rangle }_{\gamma }$ denotes nearest-neighbor (NN) sites within a honeycomb layer with bond direction *γ*, $\left\langle \left\langle {{{{{{\bf{rr}}}}}}}^{{\prime} }\right\rangle \right\rangle$ and $\left\langle \left\langle \left\langle {{{{{{\bf{rr}}}}}}}^{{\prime} }\right\rangle \right\rangle \right\rangle$ denote pairs of second-NN and third-NN, respectively, and ${\left\langle {{{{{{\bf{rr}}}}}}}^{{\prime} }\right\rangle }_{c}$ denotes NN sites between two neighboring honeycomb layers. The exchange constants ${\tilde{J}}_{H1,{{{{{\bf{r}}}}}}{{{{{{\bf{r}}}}}}}^{{{{\prime} }}}}$, ${J}_{H2}$, ${J}_{H3}$, ${J}_{K}$, and ${J}_{D}$ quantify NN Heisenberg, second-NN Heisenberg, third-NN Heisenberg, Kitaev, and Dzyaloshinskii–Moriya (DM) interactions, respectively, with the DM (unit) vectors ${\hat{{{{{{\bf{D}}}}}}}}_{{{{{{\bf{rr}}}}}}{\prime} }$ as specified in ref. ^37^. The inter-layer exchange constant ${J}_{{Hc}}$ quantifies the interaction between neighboring layers of the honeycomb lattice. The out-of-plane single-ion anisotropy is given by

3 $${H}_{{{{{{\rm{si}}}}}}}={J}_{A}\mathop{\sum}\limits_{{{{{{\bf{r}}}}}}}{\left({S}_{{{{{{\bf{r}}}}}}}^{x}+{S}_{{{{{{\bf{r}}}}}}}^{y}+{S}_{{{{{{\bf{r}}}}}}}^{z}\right)}^{2},$$

where ${J}_{A}$ is the anisotropy energy. The ${A}_{1{{{{{\rm{g}}}}}}}^{2}$ lattice distortion modifies the Heisenberg exchange as ${\tilde{J}}_{H1,{{{{{\bf{r}}}}}}{{{{{{\bf{r}}}}}}}^{{{{\prime} }}}}={J}_{H1}\left(1+\phi \left({X}_{{{{{{\bf{r}}}}}}}+{X}_{{{{{{{\bf{r}}}}}}}^{{{{\prime} }}}}\right)/2\right)$, with spin-phonon coupling parameter *ϕ*. We neglect phonon coupling to the remaining exchange terms, since they are small in comparison. Guided by previous ab initio calculations^38–40^, we fix $({J}_{H1},{J}_{H2},{J}_{H3},{J}_{K},{J}_{D},{J}_{A},{J}_{{Hc}})=\left(-2{{{{\rm{.}}}}}4,-0{{{{\rm{.}}}}}{{{{\mathrm{1,0}}}}}{{{{\rm{.}}}}}1{{{{\rm{,}}}}}0{{{{\rm{.}}}}}9,-0{{{{\rm{.}}}}}2,-0{{{{\rm{.}}}}}2,-0{{{{\rm{.}}}}}59\right)\,{{{{\rm{meV}}}}}$ to capture the spin-wave dispersion^16,37^ and the ordering temperature ${T}_{c}$ (see the SI for more details). Setting $\phi = 1.98\,{\AA}^{-1}$ then reproduces our DFT results for $\partial \varDelta E / \partial X$. For $T \ll {T}_{c}$, our model predicts an equilibrium displacement $\left\langle X \right\rangle \sim6 \times {10}^{-4}\, \AA$, comparable to the experimentally estimated magnetostriction^41^. Table 1 summarizes the quantities relevant to the dynamical spin-phonon model and static spin exchange parameters.

**Table 1:** Dynamic spin-phonon model properties and static spin exchange parameters.

Quantity or Parameter	Variable Label	Value
Phonon mass	*M*	$6.3\times {10}^{-25}\,{{{{{\rm{kg}}}}}}$
Phonon frequency	Ω	$3.87\,{{{{{\rm{THz}}}}}}$
Spin-phonon coupling	*ϕ*	$1.98\,{\AA }^{-1}$
Dynamically modulated Heisenberg interaction	${\tilde{J}}_{H1,{{{{{\bf{rr}}}}}}^{{{\prime} }}}$	${J}_{H1}\left(1+\phi \left({X}_{{{{{{\bf{r}}}}}}}+{X}_{{{{{{{\bf{r}}}}}}}^{{{{\prime} }}}}\right)/2\right)$
Nearest neighbor Heisenberg	*J*_*H*1_	$-2.4\,{{{{{\rm{meV}}}}}}$
Second-nearest neighbor Heisenberg	*J*_*H*2_	$-0.1\,{{{{{\rm{meV}}}}}}$
Third-nearest neighbor Heisenberg	*J*_*H*3_	$0.1\,{{{{{\rm{meV}}}}}}$
Nearest-neighbor Kitaev	*J*_*K*_	$0.9\,{{{{{\rm{meV}}}}}}$
Second-nearest neighbor DM	*J*_*D*_	$-0.2\,{{{{{\rm{meV}}}}}}$
Single-ion anisotropy	*J*_*A*_	$-0.2\,{{{{{\rm{meV}}}}}}$
Inter-plane nearest neighbor Heisenberg	*J*_*Hc*_	$-0.59\,{{{{{\rm{meV}}}}}}$

Using this spin-phonon model, we simulate the impact of the pump pulse as an instantaneous helicity-dependent lattice distortion, ${X}_{{{{{{\bf{r}}}}}},\pm }\left(t=0\right)={X}_{{{{{{\bf{r}}}}}}}+{\xi }_{1}(1\pm {\xi }_{2}{m}_{{{{{{\bf{r}}}}}}})$, where *m*_**r**_ is the local projection of the spin onto the equilibrium magnetization axis, and the ± sign corresponds to the ${\sigma}_{\pm}$ pump helicity. ${\xi}_{1}$ determines the overall strength of the distortion due to the Raman process, and ${\xi}_{2}$ represents its helicity dependence; the latter can arise from the spin selectivity of the helicity-dependent photoexcitation, which transiently enhances or suppresses the local Cr moment^42^. The functional form of the lattice distortion ${X}_{{{{{{\bf{r}}}}}},\pm }\left(t\right)$ can be rationalized as follows: from a mean-field perspective, the equilibrium lattice displacement scales as ${X}_{{{{{{\bf{r}}}}}}}=\alpha {m}_{{{{{{\bf{r}}}}}}}^{2}$, for some proportionality constant *α* (see the SI for more details). Assuming that the pump pulse induces a pump helicity-dependent magnetization change ±*δ*, the phonon equilibrium position is shifted to ${X}_{{{{{{\bf{r}}}}}},\pm }^{{\prime} }=\alpha {({m}_{{{{{{\bf{r}}}}}}}\pm \delta )}^{2}$, which is a special case of the aforementioned functional form, with ${\xi }_{1}=\alpha {\delta }^{2}$ and ${\xi }_{2}=2/\delta$, that only depends on the single parameter *δ* (see the SI for details).

**Fig. 4:**
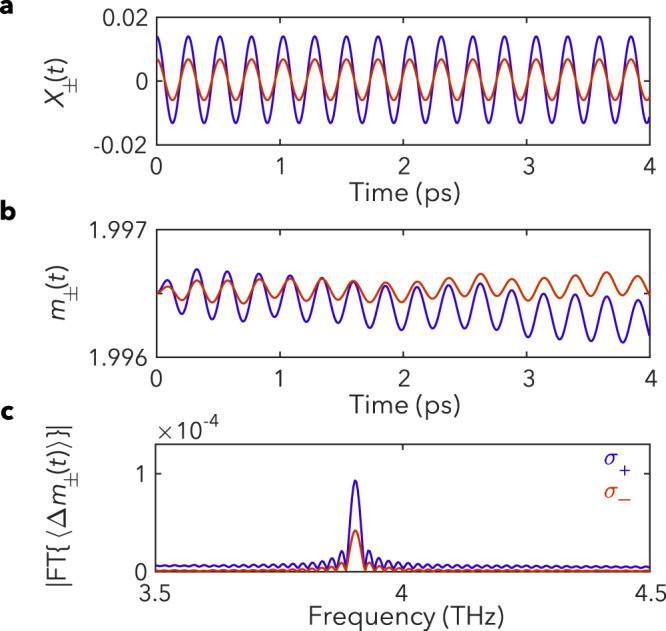
Spin-phonon dynamic simulations. Simulations at $T \, < \, {T}_{c}$, for ${\sigma }_{+}$ (blue) and ${\sigma }_{-}$ (red) pump polarizations, which show **a** coherent oscillations in the average phonon displacement, ${X}_{\pm }\left(t\right)$, **b** coherent oscillations in the magnetization, ${m}_{\pm }\left(t\right)$, driven through spin-phonon coupling, and **c** the Fourier transform of the magnetization dynamics, displaying a distinct peak at the phonon frequency.

We solve for the subsequent dynamics of the spin-phonon system by numerically integrating the coupled equations of motion (see Methods). For $T\ > \ {T}_{c}$, we find persistent oscillations in the average phonon displacement ${X}_{\pm }\left(t\right)=\frac{1}{N}{\sum }_{{{{{{\bf{r}}}}}}}{X}_{{{{{{\bf{r}}}}}},\pm }(t)$ at the phonon frequency Ω, while the uniform magnetization ${m}_{\pm }\left(t\right)$ does not exhibit coherent dynamics (see Supplementary Fig. 9d). Remarkably, in the FM phase ($T \, < \ {T}_{c}$), the distortion leads to coupled, coherent oscillations in ${X}_{\pm }\left(t\right)$ and the magnetization ${m}_{\pm }\left(t\right)$ at Ω, as shown in Fig. 4. The coherent ${m}_{\pm }\left(t\right)$ oscillations lead to oscillations in the polarization rotation, creating an additional temperature and helicity-dependent contribution to the TRPR signal below ${T}_{c}$. Setting ${\xi }_{2}=0{{{{\rm{.}}}}}18{{{{\rm{/}}}}}{\mu }_{{{\rm{B}}}}$, we find that ${m}_{+}\left(\Omega \right)/{m}_{-}\left(\Omega \right)$ plotted vs. $T/{T}_{c}$ shows excellent agreement with the experimental TRPR ratio below ${T}_{c}$ (Fig. 3c). Furthermore, in our model, the choice of ${\xi}_{2}$ also fixes the overall distortion strength, ${\xi }_{1}=0.01\,\AA$. We can use this to calculate that the resulting coherent oscillations in the magnetization are of magnitude $\varDelta {m}_{\sigma }(t)/m \sim {{{{{\mathscr{O}}}}}}({10}^{-4})$ (c.f. Fig. 4b), consistent with our experimental observations.

## Discussion

Ultimately, the overarching physical mechanism underlying the helicity-dependent response of the ${A}_{1{{{{{\rm{g}}}}}}}^{2}$ mode centers upon the coherent coupling between the magnetization and the ${A}_{1{{{{{\rm{g}}}}}}}^{2}$ phonon. Above ${T}_{c}$, the femtosecond optical pump drives transitions involving the Cr-like ${e}_{{{{{{\rm{g}}}}}}}$ level. The partial occupation of this level leads to a strongly Jahn-Teller active ion, causing the system to undergo an ultrafast trigonal distortion that triggers the coherent phonon mode. We observe these oscillations above ${T}_{c}$ in the polarization sensitive detection configuration due to the imperfect cancellation between the *p*- and *s*-polarized components of the probe pulse, an effect that has been observed for ${A}_{1{{{{{\rm{g}}}}}}}$ phonons in other systems^43,44^. This gives us a baseline signal in the non-magnetic phase that serves as a comparison to the response below ${T}_{c}$. Below ${T}_{c}$, the modulation of the spin exchange by this phonon mode leads to the intertwining of lattice vibrations and coherent spin oscillations. Moreover, symmetry-allowed virtual transitions from strongly iodine 5*p*-like states (e.g., ${t}_{1{{{{{\rm{u}}}}}}}$ and ${t}_{2{{{{{\rm{u}}}}}}}$, see Fig. 1c), which occur simultaneously with the real ${t}_{2{{{{{\rm{g}}}}}}}$ to ${e}_{{{{{{\rm{g}}}}}}}$ transitions described earlier, lead to helicity-dependent changes in the local single ion moment, transiently enhancing (suppressing) it under ${\sigma }_{+}$ (${\sigma }_{-}$) photoexcitation in a majority spin-up photoexcited volume due to optical selection rules. For example, for a spin-up domain, a right circularly polarized pulse would excite a spin-up electron from the ${t}_{1{{{{{\rm{u}}}}}}}$ or ${t}_{2{{{{{\rm{u}}}}}}}$ level to the partially occupied ${e}_{{{{{{\rm{g}}}}}}}$ level. This transiently enhances the local single-ion moment, prior to the system returning to the ground state through phonon emission. As shown above, this contributes a helicity-dependent component to the impulsive lattice distortion, akin to a dynamic magnetostriction process. Accordingly, this gives rise to a measured oscillatory signal that is an admixture of the non-temperature-dependent vibrational contribution (analogous to the $T \, > \ {T}_{c}$ case) and a temperature-dependent polarization modulation driven by the magneto-optical Kerr effect through spin-phonon coupling. While there is no symmetry constraint preventing this from also occurring for the ${A}_{1{{{{{\rm{g}}}}}}}^{1}$ mode, our DFT calculations reveal that the exchange modulation is approximately a factor of 2 smaller than for the ${A}_{1{{{{{\rm{g}}}}}}}^{2}$ mode. Therefore, we hypothesize that the lack of appreciable helicity dependence for this mode is due to the combined effects of the smaller spin-phonon coupling and smaller lattice distortion associated with the ${A}_{1{{{{{\rm{g}}}}}}}^{1}$ mode as compared to the ${A}_{1{{{{{\rm{g}}}}}}}^{2}$ mode, which can be inferred from the smaller Raman peak intensity observed via spontaneous Raman scattering^31^ and its comparatively smaller oscillatory amplitude, as observed in our experiments.

Our work demonstrates several key findings in the burgeoning field of the ultrafast control of vdW magnets. First, the inherently strong spin-phonon coupling in these materials provides a fruitful route for the control of both the vibrational and magnetic dynamics via photon helicity, allowing for a new degree of control over their transient properties. Second, while previous spontaneous Raman results required resonant excitation, our observation of coherent spin-phonons driven via ISRS with photons well below the dominant ligand-metal charge transfer resonances demonstrates that femtosecond optical spectroscopy may be a significantly more sensitive probe of vibrational and magnetic excitations in CrI_3_. Moreover, it may provide a pathway to more completely characterize the vibrational modes of CrI_3_, especially with regard to differences in the tensors governing stimulated and spontaneous Raman scattering from phonons^45,46^. Finally, our observation of an additional $3.73\,{{{{\rm{THz}}}}}$ vibrational mode in bulk CrI_3_ is in stark contrast to past spontaneous Raman measurements, where it was only observed in few-layer flakes. The persistence of this mode in thick samples, which was previously linked exclusively to near-surface AFM order in the few-layer crystal^30,33^, suggests that it is far more robust than previously believed. Moreover, in our experiments, any sort of a near-surface AFM region would make up an extremely small percentage of the overall photoexcited volume due to the large optical penetration depth, belying the clear signature of this mode below ${T}_{c}$. Instead, our simultaneous observation of modes linked to both FM and AFM order in truly bulk samples is in excellent agreement with a recent report demonstrating the coexistence of monoclinic and rhombohedral structural phases (Fig. 1a) in bulk CrI_3_ over a wide temperature range^47^. Together with our results, this suggests that FM and AFM exchange couplings and phases may be present throughout the volume of the crystal^47^, rather than restricted to the near-surface region, as was previously hypothesized.

We note that the implied magnetization changes in our theoretical picture are in excess of the saturation magnetization, suggesting that the microscopic mechanisms which drive the lattice displacement are not fully captured by our phenomenological model. Their precise nature thus remains to be uncovered and would be a fruitful avenue for future research. Another crucial area of future experimental efforts will be to study the impact of sample thickness on non-equilibrium magnetization dynamics, which would be critical for future low-dimensional device applications. Finally, a fundamental challenge to overcome is the relatively low damage threshold of CrI_3_, especially under focused ultrafast illumination, which makes fluence-dependent measurements a distinct challenge. This may be circumvented, however, by driving the system at longer wavelengths, where the absorption is significantly lower. Here, nonlinear effects are easier to drive, due to the ability to use higher pump fluences, and may prove successful in more efficiently inducing transient magnetic phenomena in CrI_3_.

In conclusion, our measurements and simulations highlight the inextricable link between magnetic order and structure in CrI_3_, due to the intimate relationship between the strength of exchange interactions, trigonal lattice distortions, and the orbital character of the states involved in the photoexcitation process. This in turn allows for greater flexibility in the manipulation of magnetization and vibrational dynamics in vdW materials, especially using all-optical techniques that exploit nonlinear processes to drive coherent phenomena. Finally, our results shed new light on the immense potential for CrI_3_ and similar 2D magnetic materials in the next generation of opto-magnetic technologies and provide insight into enabling ultrafast optical control of magnetism at the atomic limit.

## Methods

### Time-resolved experiments

Bulk-like flakes of CrI_3_, grown using chemical vapor transport, were deposited onto an Au coated sample holder and placed in a variable temperature liquid helium flow optical cryostat. Ultrafast pulses were supplied by a regeneratively amplified Ti:Sapphire laser, which generated $1.55\,{{{{\rm{eV}}}}}$ pulses ($800\,{{{{\rm{nm}}}}}$ central wavelength) with an $85\,{{{{\rm{fs}}}}}$ duration (measured at the sample position) at a $100\,{{{{\rm{kHz}}}}}$ repetition rate. The beam was split into pump and probe arms using a plate beam splitter. The pump beam was sent through a mechanical delay line, and both beams were passed through achromatic wave plates and subsequently focused onto optically flat regions of the sample at near-normal incidence using a 20X near-IR optimized apochromatic objective. The nominal $1/{e}^{2}$ focal spot diameter of the probe was approximately 10–15 μm, and the pump was approximately 20 μm, yielding a pump fluence of ${\sim} 0.64\,{{{{\rm{mJ/cm}}}}}^{2}$. The latter was just below the damage threshold of the sample (i.e., yielding no observable signal degradation or photodarkening of the sample), while also being the lowest fluence we could utilize while still maintaining a sufficient ratio with respect to the probe fluence. We performed experiments where the pump polarization was horizontally polarized (i.e., parallel to the optical table surface), right circularly polarized, and left circularly polarized. The probe beam was linearly polarized, and upon reflection, the beam was decomposed into its two orthogonal linear components using a Wollaston prism. These components were then differentially detected using a balanced Si photodiode detector and the output signal was fed into a lock-in amplifier to isolate the TRPR signal. Both the pump and probe beams were modulated using a 7/5 slotted optical chopper, allowing the lock-in amplifier to be referenced to the sum inter-modulation frequency ($1.2\,{{{{\rm{kHz}}}}}$) to eliminate pump scattering effects.

### Density functional theory calculations

Our ab initio simulations were carried out within density functional theory. We used both the pseudopotential plane wave method implemented in the Vienna ab initio simulation package (VASP)^48,49^ and in Quantum Espresso (QE), and the full-potential all-electron linearized augmented plane wave (FP-LAPW) method implemented in the Wien2k code^50^. The local density approximation was used for the exchange-correlation functional throughout all our ab initio simulations. For the VASP calculations, we chose an energy cutoff of $500\,{{{{\rm{eV}}}}}$ for the plane-wave basis set and used a 10 × 10 × 10 (10 × 10 × 3) *Γ*-centered $k$-point mesh to sample the bulk (monolayer) Brillouin zone to perform the structure optimization, *Γ*-point phonon, Raman tensor (aided with the vasp_raman python script^51^), and electronic structure calculations. The QE calculations used a plane-wave basis set with a cutoff energy of $60\,{{{{\rm{Ry}}}}}$. A 10 × 10 × 10 $k$-point mesh was used to calculate the electronic structure of bulk CrI_3_ and to support a calculation of the phonon dispersion at the $\varGamma$-point. In the Wien2k simulations, we focused on calculating the electronic band structure for bulk CrI_3_ by taking a 15 × 15 × 15 $k$-point mesh with a muffin-tin radius of $2{{{{\rm{.}}}}}50{a}_{0}$ (Cr) and $2{{{{\rm{.}}}}}35{a}_{0}$ (I), where ${a}_{0}$ is the Bohr radius.

We also used VASP to perform total energy calculations for the FM and AFM states of a CrI_3_ monolayer, from which a Heisenberg model with nearest-neighbor exchange interactions was fit to obtain the exchange interaction. The spin-lattice coupling strength was then determined by distorting the equilibrium lattice according to the displacement field for a chosen vibration mode. The electronic structure obtained in our simulations on both bulk and 2D CrI_3_ is consistent with those reported in the literature^28,52,53^. We note that SOC reduces the band gap. However, since SOC does not qualitatively change the lattice dynamics^28^, we did not include it in the calculation of the Raman tensor and estimate of the spin-lattice coupling strength.

Additionally, we performed LDA+U calculations using a Hubbard parameter of ${U}_{{{{{\rm{eff}}}}}}=2.2\,{{{{\rm{eV}}}}}$ on the Cr 3$d$ orbitals^52^ to compare with the LDA calculations. Our results are in good agreement with those reported by Liu *et al*.^54^. We found the lattice constants change slightly, which suggests a minor change of phonon modes. This is consistent with the results found by similar studies^39,55^. For the electronic properties, as shown in Supplementary Fig. 13 of the SI, we found the spin down Cr-3$d$ bands are pushed to higher energy whereas the energy gap remains at about $1\,{{{{\rm{eV}}}}}$, close to that with LDA. This observation is also consistent with that reported by Liu et al. for monolayer CrI_3_^54^. This band structure change could affect the photoinduced carrier density and the initial relaxation. However, it is not likely to change the long-time recombination and relaxation, and the physics of the spin-lattice coupling driven relaxation process.

### Monte Carlo simulations

We performed classical Monte Carlo simulations on the coupled spin-phonon system described by the Hamiltonian $H={H}_{{{{{{\rm{ph}}}}}}}+{H}_{{{{{{\rm{sp}}}}}}}$ specified in Eqs. (1)-(3) to generate a thermal ensemble of states. We studied lattices of 2 × *L* × *L* × *L*_*c*_ spins, with system sizes up to $L=32$ and ${L}_{c}=8$, with periodic boundary conditions. To improve the statistical convergence of the simulations, we employed a parallel tempering scheme^56^ to simulate 144 logarithmically spaced temperature points between ${T}_{{{\min }}}\approx 9\,{{{{\rm{K}}}}}$ and ${T}_{{{\max }}}\approx 120\,{{{{\rm{K}}}}}$ in parallel. Each simulation was equilibrated for 10^6^ sweeps before taking measurements of the specific heat and the equilibrium magnetization for an additional $1.5 \times {10}^{7}$ sweeps; a single sweep is defined as one attempted update per spin or phonon degree of freedom. As shown in the SI, the chosen coupling constants reproduce the experimental spin-wave dispersion^37^. In addition, the computed temperature-dependent specific heat and magnetization yield ${T}_{c}=42.8\,{{{{\rm{K}}}}}$ which shifts to $71\,{{{{\rm{K}}}}}$ when considering quantum corrections by rescaling to the effective spin length ${S}_{{{{{{\rm{eff}}}}}}}=\sqrt{S\left(S+1\right)}$, with $S=3/2$ for Cr (see the SI for more details). This value is in reasonable agreement with the experimental result ${T}_{c}=61\,{{{{\rm{K}}}}}$.

### Dynamical Simulations

We modeled the impact of the pump laser as a helicity-dependent local lattice distortion, given by ${X}_{{{{{{\bf{r}}}}}},\pm }\left(t=0\right)={X}_{{{{{{\bf{r}}}}}}}+{\xi }_{1}\left(1\pm {\xi }_{2}{m}_{{{{{{\bf{r}}}}}}}\right)$ at time $t = 0$. Here, ${X}_{{{\bf{r}}}}$ is the equilibrium lattice displacement at lattice site **r**, ${m}_{{{\bf{r}}}}$ is the local magnetization of the spin at site **r** along the global equilibrium magnetization direction, and the sign ± distinguishes ${\sigma }_{+}$ and ${\sigma }_{-}$ polarized pump pulses. Such a distortion could arise from an ultrafast trigonal splitting of a photoexcited Jahn-Teller active Cr ${e}_{{{{{{\rm{g}}}}}}}$ level, with the splitting being sensitive to the local Cr spin due to Hund’s coupling. We applied this ultrafast distortion to individual configurations selected from our Monte Carlo ensemble with $L=32$ and ${L}_{c}=8$. The post-distortion dynamics were described using the Landau-Lifshitz equations for spins given by

4 $$\hslash \frac{d{S}_{{{{{{\bf{r}}}}}}}^{x}}{dt}=	 \mathop{\sum}\limits_{\gamma =x,y,z}{\tilde{J}}_{H1,{{{{{\bf{r}}}}}}{{{{{{\bf{r}}}}}}}_{\gamma }}\big({S}_{{{{{{\bf{r}}}}}}}^{z}{S}_{{{{{{{\bf{r}}}}}}}_{\gamma }}^{y}-{S}_{{{{{{\bf{r}}}}}}}^{y}{S}_{{{{{{{\bf{r}}}}}}}_{\gamma }}^{z}\big)+{J}_{K}\big({S}_{{{{{{\bf{r}}}}}}}^{z}{S}_{{{{{{{\bf{r}}}}}}}_{y}}^{y}-{S}_{{{{{{\bf{r}}}}}}}^{y}{S}_{{{{{{{\bf{r}}}}}}}_{z}}^{z}\big)\\ 	+\mathop{\sum}\limits_{{{{{{\bf{r}}}}}}^{\prime} }\big[{J}_{H2}\big({S}_{{{{{{\bf{r}}}}}}}^{z}{S}_{{{{{{\bf{r}}}}}}^{\prime} }^{y}-{S}_{{{{{{\bf{r}}}}}}}^{y}{S}_{{{{{{\bf{r}}}}}}^{\prime} }^{z}\big)+{J}_{D}\,{D}_{{{{{{\bf{rr}}}}}}^{\prime} }^{x}\big({S}_{{{{{{\bf{r}}}}}}}^{y}\big({S}_{{{{{{\bf{r}}}}}}^{\prime} }^{y}-{S}_{{{{{{\bf{r}}}}}}^{\prime} }^{x}\big)+{S}_{{{{{{\bf{r}}}}}}}^{z}\big({S}_{{{{{{\bf{r}}}}}}^{\prime} }^{z}-{S}_{{{{{{\bf{r}}}}}}^{\prime} }^{x}\big)\big)\big]\\ 	+\mathop{\sum}\limits_{{{{{{\bf{r}}}}}}^{\prime\prime} }{J}_{H3}\big({S}_{{{{{{\bf{r}}}}}}}^{z}{S}_{{{{{{\bf{r}}}}}}^{\prime\prime} }^{y}-{S}_{{{{{{\bf{r}}}}}}}^{y}{S}_{{{{{{\bf{r}}}}}}^{\prime\prime} }^{z}\big)+\mathop{\sum}\limits_{{{{{{{\bf{r}}}}}}}_{c}}{J}_{Hc}\big({S}_{{{{{{\bf{r}}}}}}}^{z}{S}_{{{{{{{\bf{r}}}}}}}_{c}}^{y}-{S}_{{{{{{\bf{r}}}}}}}^{y}{S}_{{{{{{{\bf{r}}}}}}}_{c}}^{z}\big)+2{J}_{A}\big({S}_{{{{{{\bf{r}}}}}}}^{z}-{S}_{{{{{{\bf{r}}}}}}}^{y}\big)\big({S}_{{{{{{\bf{r}}}}}}}^{x}+{S}_{{{{{{\bf{r}}}}}}}^{y}+{S}_{{{{{{\bf{r}}}}}}}^{z}\big),$$

with cyclic permutations of $(x,y,z)$ yielding $d{S}_{{{{{{\bf{r}}}}}}}^{y}/{dt}$ and $d{S}_{{{{{{\bf{r}}}}}}}^{z}/{dt}$ coupled with Newton’s equations for the lattice

5a $$\frac{d{X}_{{{{{{\bf{r}}}}}}}}{{dt}}=\frac{{P}_{{{{{{\bf{r}}}}}}}}{M},$$

and

5b $$\frac{d{P}_{{{{{{\bf{r}}}}}}}}{{dt}}=-M{\Omega }^{2}{X}_{{{{{{\bf{r}}}}}}}-\frac{\partial {H}_{{{{{{\rm{sp}}}}}}}}{\partial {X}_{{{{{{\bf{r}}}}}}}}.$$

In Eq. (4), ${{{\bf{r}}}}_{\gamma }$ denotes the NN lattice site of **r** along the in-plane bond direction *γ* and ${{{\bf{r}}}}^{\prime}$ (${{{\bf{r}}}}^{\prime\prime}$) denotes the second (third) in-plane NN; the inter-plane NN direction is denoted by ${{{\bf{r}}}}_{c}$. These equations were numerically integrated using a ninth-order Runge-Kutta algorithm^57^ and adaptive time steps with a local relative error tolerance of ${\varepsilon }_{{{{{{\rm{rel}}}}}}}={10}^{-13}$. This yielded converged results up to timescales of approximately $50\,{{{\rm{ps}}}}$ in the magnetically ordered phase. We averaged the resulting dynamical observables over 10000 Monte Carlo configurations with positive magnetization (when projected onto the twofold degenerate polarization axis). In particular, we extracted the time-dependent deviation of the magnetization, $\Delta {m}_{\pm }\left(t\right)=\langle {m}_{\pm }\left(t\right)\rangle -m$, where *m* is the mean magnetization in thermal equilibrium and $\langle {m}_{\pm }\left(t\right)\rangle$ is the time-dependent magnetization after the ultrafast ${\sigma }_{\pm}$-helicity-dependent distortion was applied. We determined the ratio of Fourier components ${m}_{+}\left(\Omega \right)/{m}_{-}\left(\Omega \right)$ by calculating the Fourier transform ${m}_{\pm }\left(\omega \right)={{{{{\rm{FT}}}}}}\left\{\Delta {m}_{\pm }\left(t\right)\right\}$ and fitting a Gaussian profile to the peak found at the ${A}_{1{{{{{\rm{g}}}}}}}^{2}$ phonon frequency Ω. The ratio ${m}_{+}\left(\Omega \right)/{m}_{-}\left(\Omega \right)$ is independent of the overall distortion ${\xi}_{1}$, while a helicity-dependent splitting ${\xi }_{2}\approx 0{{{{\rm{.}}}}}18{{{{\rm{/}}}}}{\mu }_{{{\rm{B}}}}$ was found to reproduce our experimental results below ${T}_{c}$. Additional results on the time-resolved magnetization and phonon displacement are shown in the SI. Finally, our results are qualitatively robust upon tuning the Hamiltonian parameters, as long as the Kitaev exchange remains finite. In particular, our results also hold for a single-layer honeycomb model as well as for an alternate model with dominant Kitaev interactions, which has been proposed in Ref. ^16^.

## Supplementary information

Supplementary Information

## Data Availability

The data that support the findings of this study are available from the corresponding authors upon reasonable request, in order to comply with Los Alamos National Laboratory policy on data security.

## Code availability

The Vienna ab initio simulation package is available at https://www.vasp.at. The Quantum Espresso package is available at https://www.quantum-espresso.org. The Wien2k package is available at http://susi.theochem.tuwien.ac.at. The Monte Carlo and dynamical simulation codes are available from the corresponding authors upon reasonable request.
