# Supplementary material for: Coherent helicity-dependent spin-phonon oscillations in the ferromagnetic van der Waals crystal CrI3
Source: Nat Commun. 2022 Aug 2;13:4473. doi: 10.1038/s41467-022-31786-3 (PMC9345964; doi:10.1038/s41467-022-31786-3)
Supplement: Supplementary file 1 — Supplementary Information [file 41467_2022_31786_MOESM1_ESM.pdf]

Supplementary information: Coherent helicity-dependent spin-phonon oscillations in the ferromagnetic van der Waals crystal CrI_3

P. Padmanabhan[1,†,*], F. L. Buessen[2,†], R. Tutchton[1], K. W. C. Kwock[1,3], S. Gilinsky[1], M. C. Lee[1], M. A. McGuire[4], S. R. Singamaneni[5], D. A. Yarotski[1], A. Paramekanti[2,*], J.-X. Zhu[1,*], R. P. Prasankumar[1,6,*]

[1]Center for Integrated Nanotechnologies, Los Alamos National Laboratory, Los Alamos, NM 87545, USA

[2]Department of Physics, University of Toronto, Toronto, Ontario M5S 1A7, Canada

[3]The Fu Foundation School of Engineering and Applied Science, Columbia University, New York, NY 10027, USA

[4]Materials Science and Technology Division, Oak Ridge National Laboratory, Oak Ridge, TN 37831, USA

[5]Department of Physics, The University of Texas at El Paso, El Paso, TX 79968, USA

[6]Deep Science Fund, Intellectual Ventures, Bellevue, WA 98005, USA

† Authors contributed equally

[*] Authors to whom correspondence should be addressed: prashpad@lanl.gov, arunp@physics.utoronto.ca, jxzhu@lanl.gov, rpprasan@lanl.gov

The Mott Density Criterion

To estimate the photoexcited carrier density, we begin by estimating the relevant optical constants of CrI_3. The complex dielectric functions and associated complex refractive index for right circularly polarized (σ_+) and left circularly polarized (σ_-) light can be computed from the linear dielectric response functions as

$$\tilde{\epsilon}_\pm = \tilde{\epsilon}_{xx} + i\tilde{\epsilon}_{xy}, \text{ and} \tag{1}$$

$$\tilde{n}_\pm = \sqrt{\tilde{\epsilon}_{xx} + i\tilde{\epsilon}_{xy}}. \tag{2}$$

This allows us to obtain the normal incidence reflectivity and absorption coefficients as

$$R_\pm = \left|\frac{\tilde{n}_\pm - 1}{\tilde{n}_\pm + 1}\right|^2, \text{ and} \tag{3}$$

$$\alpha_\pm = \frac{4\pi}{\lambda}\Im\{\tilde{n}_\pm\}. \tag{4}$$

Assuming a spin-up domain and using the calculated linear dielectric functions[1], we estimate the σ_+ reflectivity and absorption coefficient at $E_\mathrm{p} = 1.55$ eV to be approximately $R_+ = 0.148$ and $\alpha_+ = 1.81\times10^4$ cm^{-1}, respectively. In contrast, for σ_- photons, $R_- = 0.135$ and $\alpha_- = 2.87\times10^2$ cm^{-1}, highlighting the dramatically larger absorption of σ_+ light in spin-up domains. Despite the lack of ellipsometry data for CrI_3 in this energy range below the Curie temperature (T_c), we note that differential reflectivity measurements made on bulk-like flakes[2] yield absorption coefficients that are reasonably close to the calculated values.

Using the above parameters, we can estimate the electron-hole pair density that is photoexcited as a function of thickness to be[3]

$$N_\pm(z) = \frac{\alpha_\pm(1-R_\pm)F}{E_\mathrm{p}} e^{-\alpha_\pm z}, \tag{5}$$

where F is the fluence of the ultrafast pump pulse. Given our ~20 µm spot diameter and

photoexcitation depth of ∼550 nm[1], we calculate a photoexcited carrier density of $N_{+} = 2.50\times10^{19}$ cm^{-3} and $N_{-} = 6.33\times10^{17}$ cm^{-3}. Given the nanometer-scale exciton radius of van der Waals materials, the former density is likely to be large enough to exceed the Mott density criterion of $n_c = (0.25/a_B^*)^3$, yielding a transient photoinduced quasi-metallic state. This supports our interpretation of the demagnetization dynamics under the microscopic three-temperature model, typically applied to the case of ferromagnetic metals.

Microscopic Three-Temperature Model

We model the experimentally measured demagnetization dynamics using the microscopic three-temperature model (M3TM), which considers the impact of both conventional electron-phonon scattering and Elliott-Yafet processes that are enabled by spin orbit coupling (SOC). Here, the majority- (⇑) and minority-spin (⇓) Bloch states ($|\psi\rangle$) are comprised of admixtures of pure spin states $|\uparrow\rangle$ and $|\downarrow\rangle$, such that[4]

$$\left|\psi_{\mathbf{k}n}^{\Uparrow}\right\rangle = a_{\mathbf{k}n}^{\uparrow}|\uparrow\rangle + b_{\mathbf{k}n}^{\uparrow}|\downarrow\rangle, \text{ and} \tag{6}$$

$$\left|\psi_{\mathbf{k}n}^{\Downarrow}\right\rangle = a_{\mathbf{k}n}^{\downarrow}|\downarrow\rangle + b_{\mathbf{k}n}^{\downarrow}|\uparrow\rangle, \text{ and} \tag{7}$$

where $\mathbf{k}$ is the wave vector, n is the band index, and $a_{\mathbf{k}n}^{\uparrow,\downarrow}$ and $b_{\mathbf{k}n}^{\uparrow,\downarrow}$ are the wave function coefficents associated with the pure spin-states. Here, $b_{\mathbf{k}n}^{\uparrow,\downarrow}$ are non-zero only if SOC is present, which allows for a finite probability of phonon-mediated spin-flip scattering with a matrix element of the form[5]

$$g_{mn\mathbf{q}}^{\Downarrow\Uparrow} = \left\langle\psi_{\mathbf{k+q}m}^{\Downarrow}\middle|\Delta V_{\mathbf{q}}\middle|\psi_{\mathbf{k}n}^{\Uparrow}\right\rangle \neq 0 \tag{8}$$

where $\mathbf{q}$ is the phonon wave vector and ΔV is the interaction potential.

This is the basis of the M3TM, which uses a system of three partial differential equations to describe the interacting electronic, phonon, and spin subsystems as[6]

$$C_e(T_e)\frac{dT_e}{dt} = \kappa\nabla_z^2 T_e + G_{ep}\left[T_p - T_e\right] - C_e(T_e)\frac{T_e - T_0}{\tau_{\mathrm{th}}} + P_0 G(t), \tag{9a}$$

$$C_p\frac{dT_p}{dt} = G_{ep}\left[T_e - T_p\right], \text{ and} \tag{9b}$$

$$\frac{dm}{dt} = Rm\frac{T_p}{T_C}\left[1 - m\coth\left(m\frac{T_c}{T_e}\right)\right], \tag{9c}$$

where T_e is the electronic temperature, T_p is the phonon temperature, and m is the magnetization. The relevant CrI_3 material properties are the thermal conductivity κ, the electronic specific heat $C_e = \gamma_e T_e$, where γ_e is the electronic specific heat coefficient, and the phonon specific heat C_p. R is a material-dependent parameter given by[6]

$$R = \frac{8a_{\mathrm{sf}}G_{ep}k_B T_c^2 V}{\mu E_D^2}, \tag{10}$$

where k_B is Boltzmann's constant, V is the formula unit volume, μ is the magnetic moment, and

Parameter	Value
κ ($Wm^{-1}K^{-1}$)	1.36
γ ($Jm^{-3}K^{-2}$)	550
C_p ($Jm^{-3}K^{-1}$)	1.5×10^{5}
V (m^3)	1.35×10^{-28}
μ (μ_B)	3.87
E_D (J)	1.85×10^{-21}
T_c (K)	61

Supplementary Table 1. M3TM material parameters. Material parameters used in the M3TM that were obtained from literature. κ was obtained from Ref. 7, γ_e, C_p, μ, E_D, and T_c were obtained from Ref. 8, and V was estimated from the material parameters in Ref. [9].

E_D is the Debye energy. G_{ep}, a_{sf}, τ_{th}, and P_0 are the electron-phonon coupling constant, the spin-flip probability, the thermal diffusion time, and the energy absorption from the Gaussian pump pulse $G(t)$, which are treated as free parameters in the fitting procedure. The material parameters obtained from the literature are summarized in Supplementary Table 1. In particular, the thermal conductivity was obtained directly from DFT calculations presented in Ref. 7. The electronic specific heat coefficient and phonon specific heat were estimated from the low temperature heat capacity, plotted as C/T vs T^2 in Fig. 6c of Ref. 8. This was converted to units of $\mathrm{Jm^{-3}K^{-3}}$ by multiplying by a conversion factor of $a = 75/2.025 = 37$ by comparison with Refs. [10] and [11]. The intercept of Fig. 6c of Ref. 8 then gives the electronic specific heat coefficient, which can be converted to $\mathrm{J/m^3K^2}$ by multiplying by the molar mass (432.7 g/mol) and the density (5.32×10^6 kg/m^3), which were obtained from Ref. [9]. The phonon specific heat was estimated in the same way from the slope of Fig. 6c of Ref. 8. The formula unit volume was approximated by dividing the molar mass by the density and Avogadro's number. The Debye energy was obtained by multiplying the Debye temperature of 134 K from Ref. 8 by Boltzmann's constant. Finally, the magnetic moment and critical temperature were obtained directly from Ref 8.

We solved the system of equations (9) over a temporal window of 400 ps and a depth into the sample of 100 nm. The fit parameters were globally optimized using an algorithm that used multiple initial start points across the parameter space and a constrained minimization process using an interior point algorithm as implemented in MATLAB[12] and a cost function consisting of the absolute difference between the calculated and experimentally obtained magnetization dynamics. This was parallelized using MATLAB's parallel computing toolbox to reduce the computation time. The partial differential equation system outlined in eq. (9) was solved using MATLAB's parabolic-elliptic partial differential equation solver which uses a variable-step,

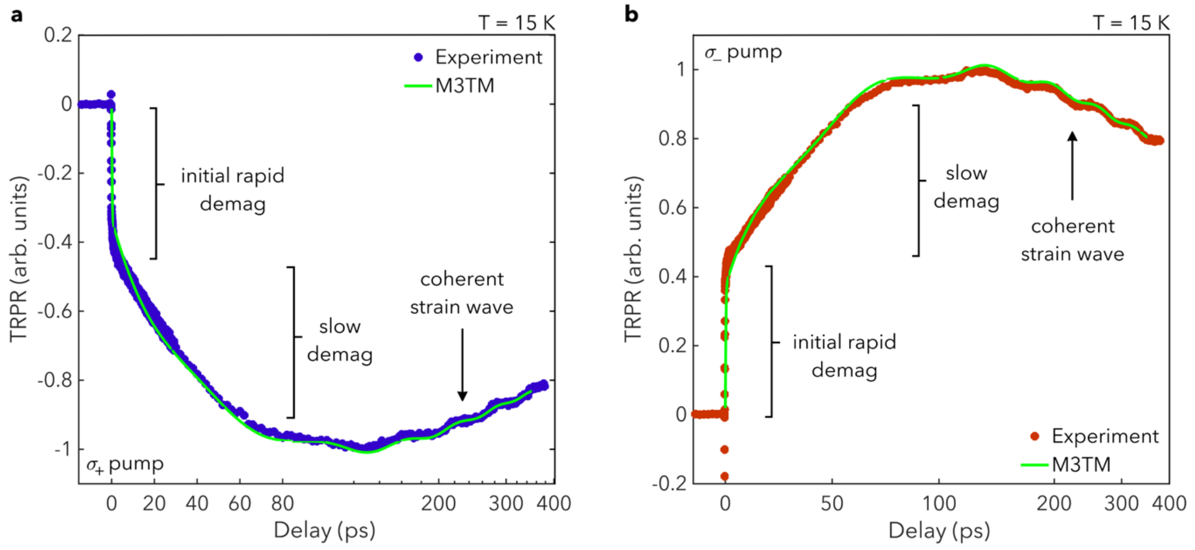

Supplementary Figure 1. Time-resolved polarization rotation signals and M3TM fits. Time-resolved polarization rotation signals T = 15 K under **a** σ_+ and **b** σ_- pumping. The colored dots are experimental data and the green traces are fits using the M3TM plus a decaying 16 GHz sinusoidal signal obtained by fitting the experimental data.

variable-order solver based on the numerical differentiation formulas of orders 1 to 5 for time integration. The resulting M3TM fits for the σ_+ and σ_- pumping conditions are indicated by the green lines in Supplementary Fig. 1 and are calculated as

$$TRPR \propto \int m(t,z)e^{-z/\delta_{\mathrm{p}}}dz\,, \quad (11)$$

where δ_{p} is the penetration depth. The fits in Supplementary Fig. 1 were obtained using fit values of $a_{\mathrm{sf}} = 0.175$, $G_{ep} = 4.05\times10^{16}$ J/m^{3}sK, $P_0 = 1.25\times10^{-5}$ J/m^{4}s, and $\tau_{\mathrm{th}} = 4$ ps for the σ_+

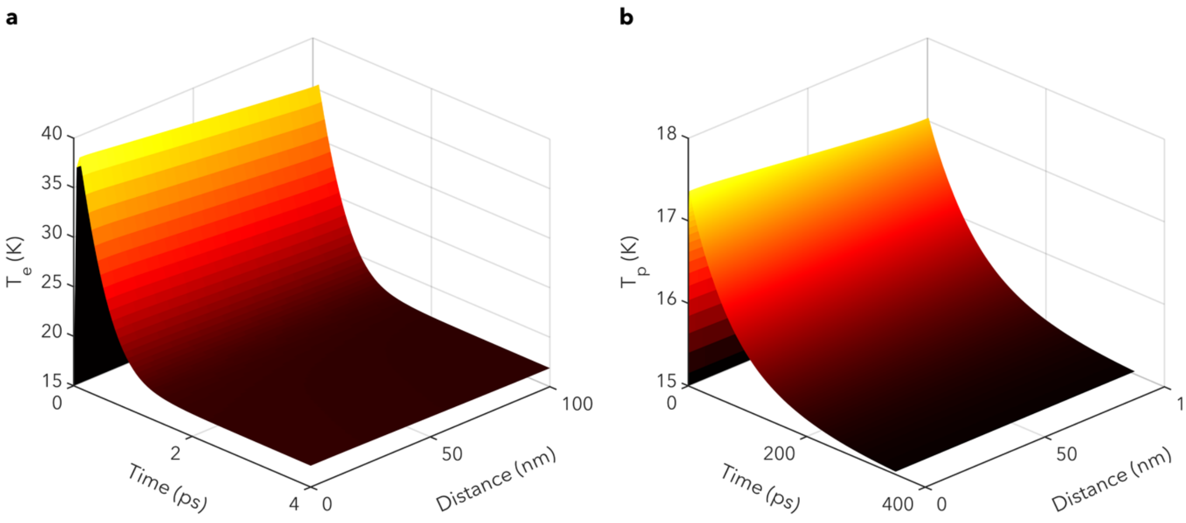

Supplementary Figure 2. Electron and phonon temperatures. a T_e and **b** T_p as a function of time and depth into the sample calculated using the M3TM under σ_+ pumping at $T = 15$ K.

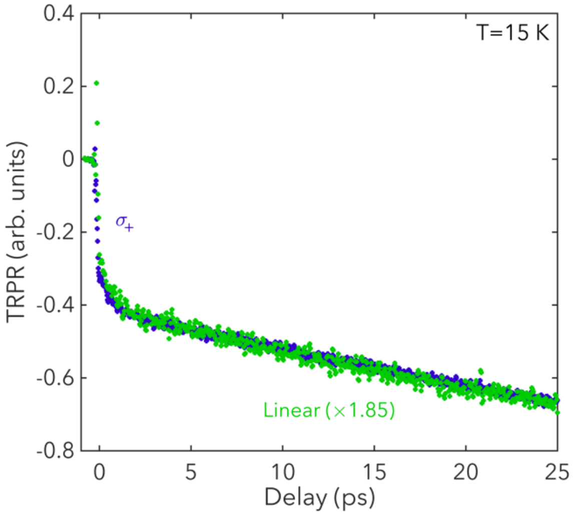

Supplementary Figure 3. Demagnetization dynamics under linear and circular pumping. Demagnetization dynamics in the first 25 ps under σ_+ (blue) and linearly (green) polarized pumping at $T = 15$ K. The linearly polarized data has been normalized by a factor of 1.85 to enable qualitative comparison with the circularly polarized data.

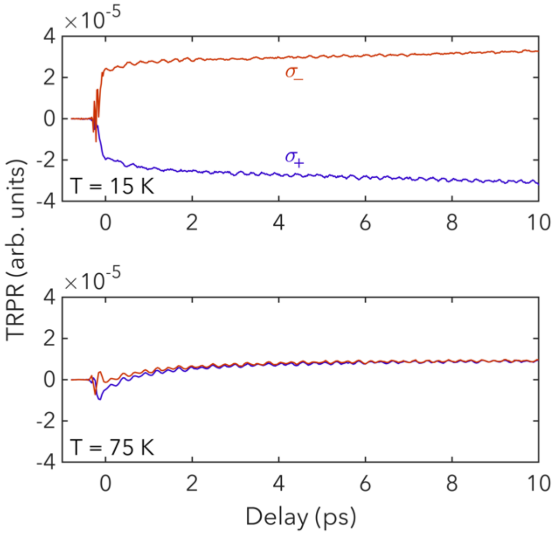

Supplementary Figure 4. Raw TRPR signals under σ_+ and σ_- pumping above and below T_C. Raw TRPR signals under σ_+ (blue) and σ_- (red) pumping below ($T = 15$ K, upper panel) and above ($T = 75$ K, lower panel) T_c.

pumping case and $a_{\mathrm{sf}} = 0.199$, $G_{ep} = 3.40\times10^{16}$ J/m^{3}sK, $P_0 = 1.28\times10^{-5}$ J/m^{4}s, and $\tau_{\mathrm{th}} =$ 4.88 ps for the σ_- pumping case. The additional oscillatory component in the M3TM fits, associated with the 16 GHz coherent strain wave, were added by using a decaying sinusoidal fit of the oscillatory components in the experimental data.

In addition, Supplementary Fig. 2 shows that the pump pulse causes a transient increase of ~22 K in T_e from the equilibrium temperature of $T_0 = 15$ K that decays back to equilibrium within the first 2 ps. In contrast, the change in the lattice temperature, T_p, upon photoexcitation is only ~2 K and takes several hundred picoseconds to recover back to equilibrium. Finally, we note that while we did not collect long time scale data under the linearly polarized pumping case, we note that the dynamics over the first ~25 ps are nearly identical to the σ_+ dynamics, as shown in Supplementary Fig. 3. Here, we have normalized the linearly polarized data to enable

qualitative comparison of the two curves (i.e., the timescales on which relaxation occurs). Accordingly, we do not expect linearly polarized pumping to yield substantively different fitting results.

Oscillatory Mode Fitting

We chose to focus on Fourier transform-based analysis of the oscillatory modes, which allowed us to directly extract the helicity-dependent ratio of A_{1g}^{1} and A_{1g}^{2} modes from the spectral weight in the vicinity of these modes with minimal data processing. This primarily involved removing the non-oscillatory background signals originating from ultrafast demagnetization below T_c or optical effects (e.g., induced birefringence), examples of which are shown in Supplementary Fig. 4 for data taken at $T = 15$ K and 75 K. We also performed fitting-based analysis of the time domain waveforms using models consisting of the sums of two exponentially decaying sinusoidal oscillators, each of the form $A_i e^{-t/\tau_i} \sin(\omega_i t + \phi_i)$. Here, the oscillatory component of the experimental signals was first bandpass filtered between 0.5 – 12 THz to remove low and high frequency noise far away from the optical phonon frequency range, which improved the fit quality. The results of the fitting for σ_+ and σ_- pumping at 15 K are shown in the upper panel of Supplementary Fig. 5a, yielding fit frequencies of approximately 2.4 THz and 3.86 THz that correspond to the A_{1g}^{1} and A_{1g}^{2} modes, respectively, in good agreement with the Fourier transform peak positions. We find that while the fit produces satisfactory results at later times, we observe a deviation from our experimental data in the first ~ 3 ps, indicated by the red shaded regions in Supplementary Fig. 5. We postulate that this is due to the presence of a weak signal associated with a Raman allowed phonons previously associated with AFM ordering[13,14] due to the coexistence of monoclinic and rhombohedral structural phases throughout the volume of the

bulk crystal, which was recently confirmed via temperature-dependent X-ray diffraction measurements[15]. To corroborate this, we fit our data

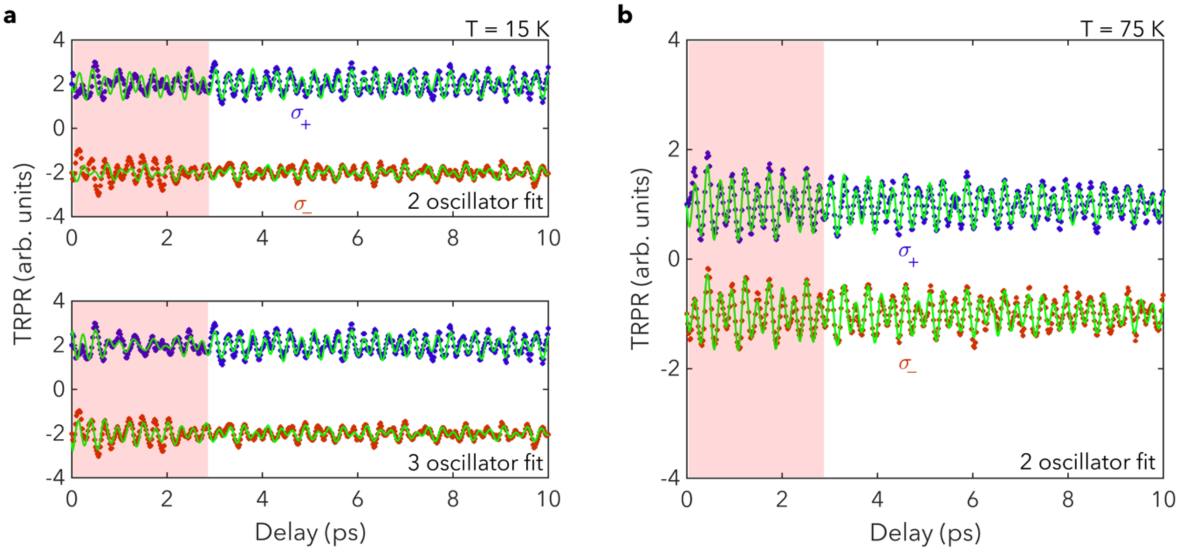

Supplementary Figure 5. Oscillator fitting under σ_+ and σ_- pumping above and below T_C. **a** Two-oscillator (upper panel) and three-oscillator (lower panel) fits (green traces) of the bandpass filtered oscillatory component of the measured TRPR signal at $T = 15$ K under σ_+ (blue dots) and σ_- (red dots) pumping and **b** two-oscillator fit (green traces) of the bandpass filtered oscillatory component of the measured TRPR signal at $T = 75$ K under σ_+ (blue dots) and σ_- (red dots) pumping. The red shaded region indicates the temporal window over which the two-oscillator model deviates from the $T = 15$ K experimental data.

with a three-oscillator model; the results for σ_+ and σ_- pumping at 15 K are shown in the lower panel of Supplementary Fig. 5a. The fit frequencies of the A_{1g}^1 and A_{1g}^2 modes remain unchanged, and the third mode fit frequency was approximately 3.73 THz. We get improved agreement in the first ~ 3 ps under both pumping configurations. Additionally, the frequency of the third oscillator is in excellent agreement with the AFM order dependent mode frequency

measured via spontaneous Raman scattering[13,14]. We also found that this mode rapidly decays (within 2 ps) and the fit amplitude was relatively insensitive to the pump polarization as compared to the A_{1g}^2 mode. In contrast, as shown in Supplementary Fig. 5b, at 75 K (above T_c) we find that the two-oscillator model captures the coherent dynamics over the full temporal window with A_{1g}^1 and A_{1g}^2 mode fit frequencies of approximately 2.4 THz and 3.85 THz, respectively. The lack of a third oscillatory mode above T_c is consistent with the absence of magnetic ordering that would be required to support an AFM order dependent mode. The fits below T_c revealed that the dominant helicity dependence was in the A_{1g}^2 mode, consistent with our Fourier transform analysis. Finally, due to the long decay time of the A_{1g}^1 and A_{1g}^2 modes below T_c, both fitting- and Fourier transform-based approaches were unable to reveal conclusive trends in the decay time of the oscillators across the phase transition.

Spin-Wave Spectrum

In our analysis we assumed the spin Hamiltonian

$$\begin{aligned} H_{\mathrm{sp}} = &\sum_{\langle \mathbf{r}\mathbf{r}'\rangle_\gamma} \left[J_{H1} \mathbf{S}_\mathbf{r} \cdot \mathbf{S}_{\mathbf{r}'} + J_K\, S_\mathbf{r}^\gamma\, S_{\mathbf{r}'}^\gamma \right] + \sum_{\langle\langle \mathbf{r}\mathbf{r}'\rangle\rangle} \mathrm{J}_{H2} \mathbf{S}_\mathbf{r} \cdot \mathbf{S}_{\mathbf{r}'} + J_D\, \widehat{\mathbf{D}}_{\mathbf{r}\mathbf{r}'} \cdot \mathbf{S}_\mathbf{r} \times \mathbf{S}_{\mathbf{r}'} \\ &+ \sum_{\langle\langle\langle \mathbf{r}\mathbf{r}'\rangle\rangle\rangle} \mathrm{J}_{H3} \mathbf{S}_\mathbf{r} \cdot \mathbf{S}_{\mathbf{r}'} + \sum_{\langle \mathbf{r}\mathbf{r}'\rangle_c} J_{Hc} \mathbf{S}_\mathbf{r} \cdot \mathbf{S}_{\mathbf{r}'} + \sum_{\mathbf{r}} J_A \left(S_\mathbf{r}^x + S_\mathbf{r}^y + S_\mathbf{r}^z \right)^2, \end{aligned} \tag{12}$$

with exchange constants $(J_{H1}, J_{H2}, J_{H3}, J_K, J_D, J_A, J_{Hc}) =$ (-2.4,-0.1, 0.1, 0.9, -0.2, -0.2, -0.59) meV (more details, including definitions of the exchange constants, etc., are in the main text). The Dzyaloshinskii-Moriya (DM) vector $\widehat{\mathbf{D}}_{\mathbf{r}\mathbf{r}'}$ assumes values $\pm\frac{1}{\sqrt{3}}(1,1,1)^T$, with the sign structure in accordance with Ref. 16. The spin Hamiltonian is guided by previous neutron scattering experiments, where it was found that the combination of Heisenberg and DM interactions, along

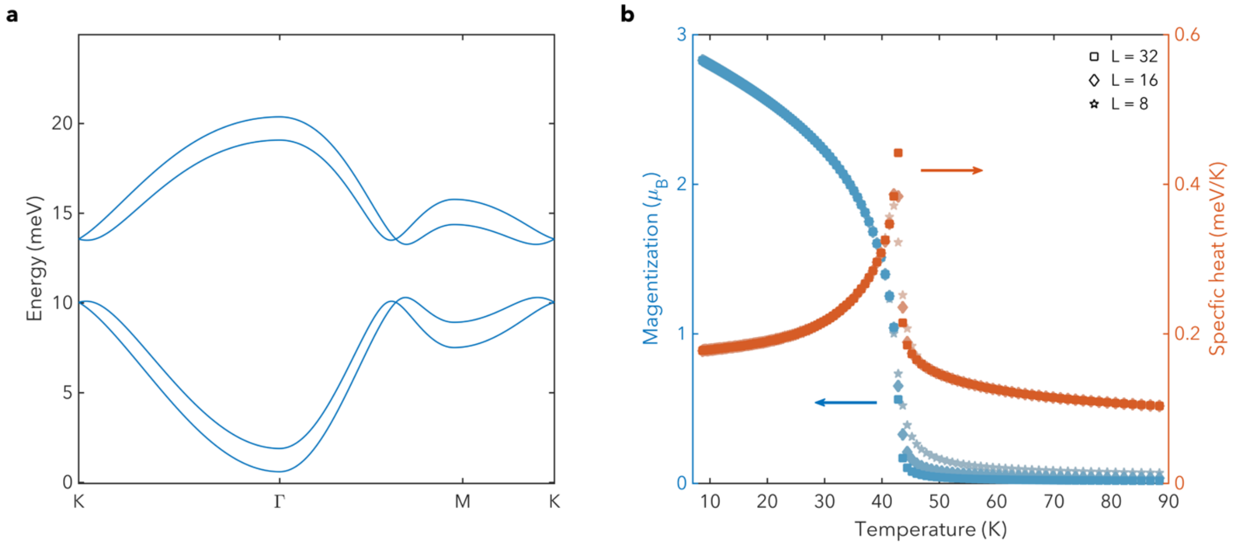

Supplementary Figure 6. Spin-waves and thermodynamics. a The spin-wave spectrum calculated from the equilibrium spin model, equation (12), captures salient features seen in experiments[17]: an overall band width ∼20 meV, a zone center excitation gap ~2 meV at the Γ-point, and a gap ∼3 meV at the BZ corners (K-points). Note that there are six bands in total, two pairs of which are degenerate. **b** Magnetization and specific heat as determined from MC simulations. The peak in the specific heat indicates the calculated magnetic ordering transition for monolayer CrI_3 at $T_c = 42.8$ K. Opaque curves were obtained at a system size $L = 32$; lighter colors indicate system sizes $L = 8$ and 16 (from lighter to darker). Statistical error bars are smaller than the plot markers.

with a local spin anisotropy, yields an excellent fit for the magnon spectrum[18]. However, the fit is not unique. It is possible to introduce finite Kitaev interactions without inducing a significant change in the magnon spectrum: on the level of nearest neighbor interactions, keeping the overall

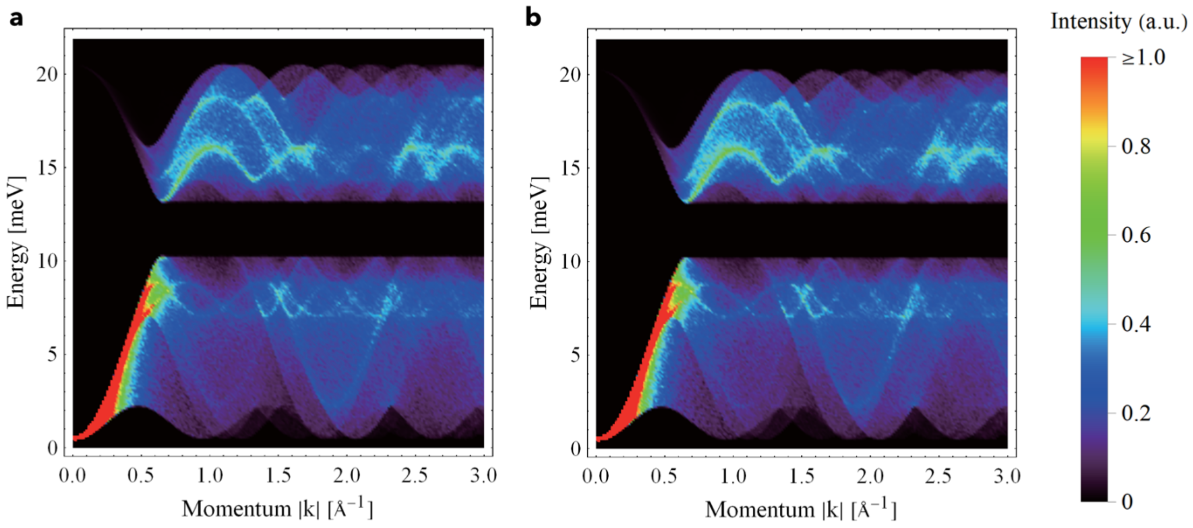

Supplementary Figure 7. Powder diffraction spectrum. a Powder diffraction spectrum for the model parameters, as determined in Ref. 18 in the absence of Kitaev exchange, and **b** model parameters used in this work with a finite Kitaev interaction. Both models provide excellent agreement with the experimentally observed magnon spectrum[18].

mean-field energy scale $J_{H1} + K/3$ constant, there is freedom to vary the ratio of Heisenberg and Kitaev contributions. In order to determine the precise ratio of Kitaev and Heisenberg interactions, we draw on previous *ab initio* calculations, which have identified the dominant role of FM Heisenberg interactions ($J_{H1} = -2.4$ meV) augmented by AFM Kitaev couplings ($K = 0.9$ meV)[19]. Remarkably, the *ab initio* study also predicted the same nearest-neighbor mean-field energy scale as the neutron scattering experiments, allowing for the two perspectives to be combined easily. The same qualitative structure of FM Heisenberg interactions in conjunction with AFM Kitaev exchange has recently also been identified in strong-coupling perturbation theory[20].

As we show in Supplementary Fig. 6a, spin-wave theory calculations for this model reproduce important features of the magnon spectrum; the spin-wave calculations are performed with a six-site unit cell (as defined in Ref. [18]), leading to a six-band spectrum. The key features include a gap at the Brillouin zone (BZ) center which is set by J_A[18], as well as the gap at the Brillouin zone corners which is set predominantly by J_D, with additional minor contributions from J_K[18]. Despite introducing finite Kitaev exchange in our model, as guided by *ab initio* theory, the overall prediction for the intensity distribution in neutron powder diffraction is in excellent agreement with the data obtained in Ref. [18] (see Supplementary Fig. 7).

Finally, we mention that a biquadratic spin exchange $(\mathbf{S_r} \cdot \mathbf{S_{r'}})^2$ has also been proposed to be a potentially relevant term in the microscopic description of CrI_3[21]. In our combined spin-phonon model, such terms would automatically emerge upon integrating out the local phonon modes, which couple to the Heisenberg spin exchange of the form $\mathbf{S_r} \cdot \mathbf{S_{r'}}$. Schematically, the terms are obtained when in a Hamiltonian of the form $H = M\Omega X^2/2 + J\Phi X S_i S_j$, where X is the phonon mode and S_i and S_j are spin operators, the phonon mode is integrated out as

$$\int DX\, e^{-H} = \int DX\, e^{-\frac{M\Omega}{2}X^2 + J\Phi X S_i S_j} \sim e^{\frac{(J\Phi S_i S_j)^2}{2M\Omega}}, \qquad (13)$$

However, those terms would scale as $J_{H1}^2 \phi^2 / M\Omega^2 \approx 10^{-3}$ meV and are therefore negligibly small within our model.

Thermodynamics: Monte Carlo Simulations

We carried out classical Monte Carlo (MC) simulations on the spin-phonon system, on layered honeycomb lattices of $2 \times L \times L \times L_c$ spins, with in-plane system sizes up to $L = 32$, the

number of layers $L_c = 8$, and periodic boundary conditions. Supplementary Fig. 6b shows the specific heat and magnetization data from our MC simulation results for system sizes $L = 8,16$, and 32 which exhibits an FM phase transition at $T_c = 42.8$ K. It is important to note that our MC simulations are purely classical, whereas the physical $S = 3/2$ spins may still be subject to quantum effects and a partial rescaling towards the quantum spin exchange energy scale $S(S + 1)$, exceeding the classical energy scale S^2 and bringing T_c closer to the experimental value. This has previously been noted for CrI_3 in Ref. [22] and incorporating such quantum corrections by rescaling to the effective spin length $S_{\text{eff}} = \sqrt{S(S+1)}$, with $S = 3/2$ for Cr shifts T_c up from its classical value by a factor of 5/3. Our classical MC simulations thus yield an enhanced $T_c(3D) \sim 71$ K, which compares favorably with the experimental bulk T_c of ~61 K. Quantum rescaling similarly manifests in other systems, for example for the $S = 1/2$ Heisenberg model on the cubic lattice, where T_c/J is found to be 0.95 in quantum Monte Carlo calculations[23], whereas the classical value is $T_c/J = 1.44/4 \approx 0.36$; this amounts to a phenomenological rescaling of 2.9, close to the value of $S(S+1)/S^2 = 3$ [24].

Phonon Coupling and Spin-Phonon Dynamics

As described in the main text, we incorporate spin-phonon coupling to the dominant Heisenberg exchange constant by $\tilde{J}_{H1,\mathbf{rr}'} = J_{H1}\left(1 + \frac{\phi}{2}(X_{\mathbf{r}} + X_{\mathbf{r}'})\right)$, with spin-phonon coupling parameter ϕ. The spin-phonon coupling may be constrained using our DFT calculations. From the model Hamiltonian, we find that the energy difference per spin between the (in-plane) AFM and FM states is given by $\Delta E = S^2(3\tilde{J}_{H1} + J_K + 3J_{H3})$, where $\tilde{J}_{H1}$ is the mean value of $\tilde{J}_{H1,\mathbf{rr}'}$ across all lattice bonds. Assuming a uniform phonon displacement X, we find $\partial\Delta E/\partial X =$

$S^2(3J_{H1}\phi)$. Choosing $\phi = 1.98$ Å^{-1} we obtain $\partial\Delta E/\partial X =$ -32 meV/Å, in agreement with our DFT estimate. For simplicity, as $J_{H2}, J_{H3}, J_K, J_D, J_A$, and J_{Hc} are already small compared to the nearest-neighbor Heisenberg exchange J_{H1}, we neglect phonon coupling to these exchange constants since it is not expected to have a significant impact on our results.

The impact of the pump pulse can be simulated as a sudden change in the local lattice displacement $X_{\mathbf{r}}$ by a combination of a magnetization-independent term and a term which is linear in the magnetization m. This is parametrized by two numbers, ξ_1 and ξ_2, according to

$$X_{\mathbf{r}} \to X'_{\mathbf{r},\sigma} = X_{\mathbf{r}} + \xi_1(1 + \sigma\xi_2 m_{\mathbf{r}}). \tag{13}$$

In our simulations, we chose $\xi_1 = 0.01$ Å; as discussed further below, however, its value does not impact the ratio of Fourier components $m_+(\Omega)/m_-(\Omega)$. Further, we found that $\xi_2 \approx 0.18/\mu_B$ led to good agreement with the experimental observation of the pump-helicity dependent asymmetry. In this supplemental section, we provide additional rationales for the functional form of the lattice distortion and its properties.

Assuming thermal equilibrium, the mean phonon displacement X can be determined by minimizing the coupled spin-phonon Hamiltonian $H = H_{\text{ph}} + H_{\text{sp}}$ with respect to X, i.e, setting $\partial H/\partial X = 0$. This leads to

$$M\Omega^2 X = -\sum_{\langle\mathbf{r}\mathbf{r}'\rangle} J_{H1}\phi\mathbf{S}_{\mathbf{r}} \cdot \mathbf{S}_{\mathbf{r}'}\,. \tag{14}$$

In the magnetically ordered phase, using a mean-field picture with average magnetization $\langle S_{\mathbf{r}}\rangle =$

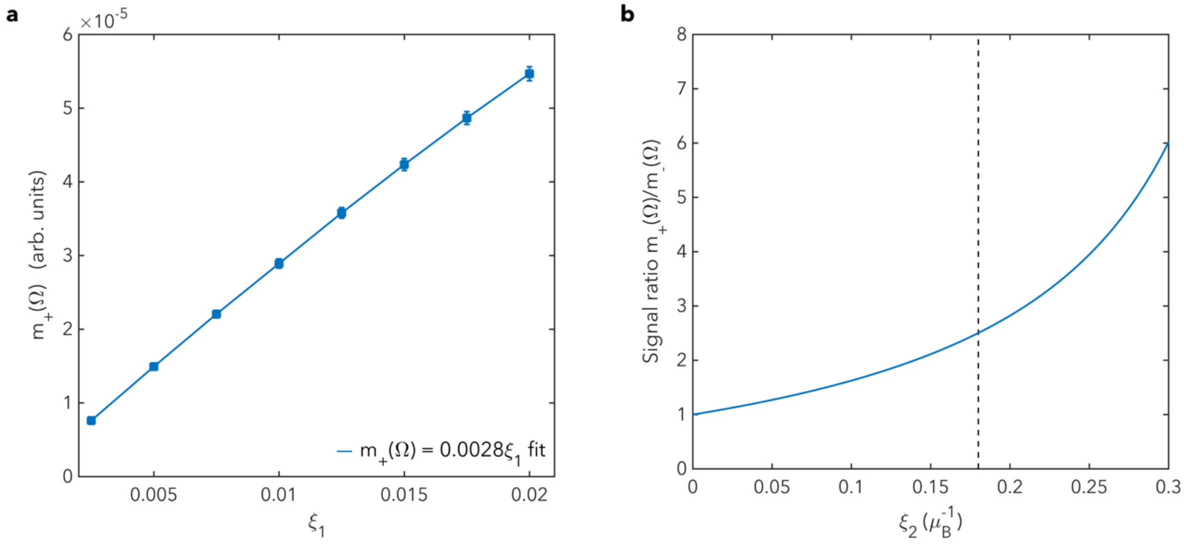

Supplementary Figure 8. Dynamical simulation parameters. a Response of the Fourier component $m_+(\Omega)$ at the phonon frequency Ω as a function of ξ_1 of the phonon (for the case $\xi_2 = 0$). Data is displayed at $T/T_c = 0.6$. It is fit by a linear function $m_+(\Omega) = 0.0028\xi_1$. **b** The Fourier component ratio $m_+(\Omega)/m_-(\Omega)$ as a function of ξ_2, based on the linear fit shown in **a**. The dashed line in **b** indicates $\xi_2 \approx 0.18/\mu_B$, as determined in the main text.

$m/(2\mu_B)$, we thus naturally find that the equilibrium phonon displacement scales quadratically with the magnetization,

$$\langle X \rangle \approx \alpha m^2, \tag{15}$$

with $\alpha = -3J_{H1}\phi/(8M\Omega^2\mu_B^2)$. This is consistent with the magnetostriction respecting time-reversal symmetry, i.e., it is invariant under $m \to -m$. At a temperature of $T/T_c = 0.6$, for example, our MC simulations indicate $\langle m \rangle \approx 2.4\mu_B$ (i.e., $\langle S_\mathrm{r} \rangle = 1.2$) and $\langle X \rangle \approx 4.9\times10^{-4}$ Å, yielding a proportionality constant of $\alpha \approx 8.5\times10^{-5}$ Å$/\mu_B^2$. This value compares well with the

mean-field picture, which, neglecting local fluctuations in the magnetization, predicts $\alpha = 7.5\times10^{-5}$ Å$/\mu_B^2$.

Given that the pump pulse is an intense non-equilibrium drive, for which we do not have a detailed microscopic model, we discuss phenomenological pictures for the pump-induced dynamics. The pump pulse can, through helicity-dependent perturbations in the local Cr moment, induce a change in magnetization by $\pm\delta$, depending on the photon polarization $\sigma = \pm 1$. This change in magnetization can impact the phonon coordinate via the relation in equation (15), leading to an additional lattice distortion component expressed as

$$\langle X'_\sigma \rangle = \alpha(m + \sigma\delta)^2 = \langle X \rangle + \alpha\delta^2 + 2\sigma\alpha m\delta, \tag{16}$$

which is of the form used in equation (13) with $\xi_1 = \alpha\delta^2$ and $\xi_2 = 2/\delta$. Such a process leads to a one-parameter description of the lattice distortion, controlled by δ.

Whereas the choice of parameters ξ_1 and ξ_2 fulfills the relation $\xi_1 = 4\alpha/\xi_2^2$ from the single-parameter description with the proportionality constant α as determined above, the magnetization change δ implied by the value of $\xi_2 \approx 0.18/\mu_B$ would be unphysically large. The one-parameter picture thus cannot be complete. An important alternative mechanism for explaining our results is based on Raman transitions induced by the incident photons via virtual intermediate state excitations from the iodine $5p$ to chromium e_g states. The intermediate singly occupied e_g level is Jahn-Teller active, which can lead to an additional trigonal lattice distortion in the Raman process. The e_g level is expected to be spin-split in the presence of magnetic order, and the photon polarization determines the spin of the virtually excited electron, which can be accounted for by a displacement $W_1 + \sigma m W_2$ (which is again invariant under simultaneous inversion of the sign of the magnetization and the incoming photon polarization). If this effect

dominates, the phonon coordinate would be modified as $\langle X'_\sigma \rangle = \langle X \rangle + W_1 + \sigma m W_2$, so that $\xi_1 = W_1$ and $\xi_2 = W_2/W_1$. In effect, ξ_1 controls the overall lattice distortion strength and the resulting coherent oscillation amplitude (averaged over pump photon helicity), while ξ_2 controls the pump-helicity dependent asymmetry in the coherent dynamics.

Modeling the lattice distortion in the manner described above robustly leads to the ratio of Fourier components $m_+(\Omega)/m_-(\Omega)$ shown in Fig. 3c of the main text. To corroborate this, we make the plot shown in Supplementary Fig. 8a, which shows that the response $m_+(\Omega)$ to an impulsive perturbation of the phonon displacement is approximately proportional to the magnitude of the strength ξ_1 (neglecting any splitting ξ_2 for simplicity).

The non-equilibrium pump-induced lattice displacement naturally affects the signal-to-noise ratio in our model calculations, such that a weaker kick is more difficult to detect unless we average over a larger number of ensemble configurations. Due to the linear scaling, the ratio of Fourier components $m_+(\Omega)/m_-(\Omega)$ is independent of ξ_1; its dependence on ξ_2 is shown in Supplementary Fig. 8b. Furthermore, the helicity-dependent asymmetry linearly depends on the magnetization. Consequently, the functional form of the ratio of Fourier components should have a similar characteristic onset as the magnetization curve below T_c, which we indeed observe, c.f. the magnetization curve in Supplementary Fig. 6b.

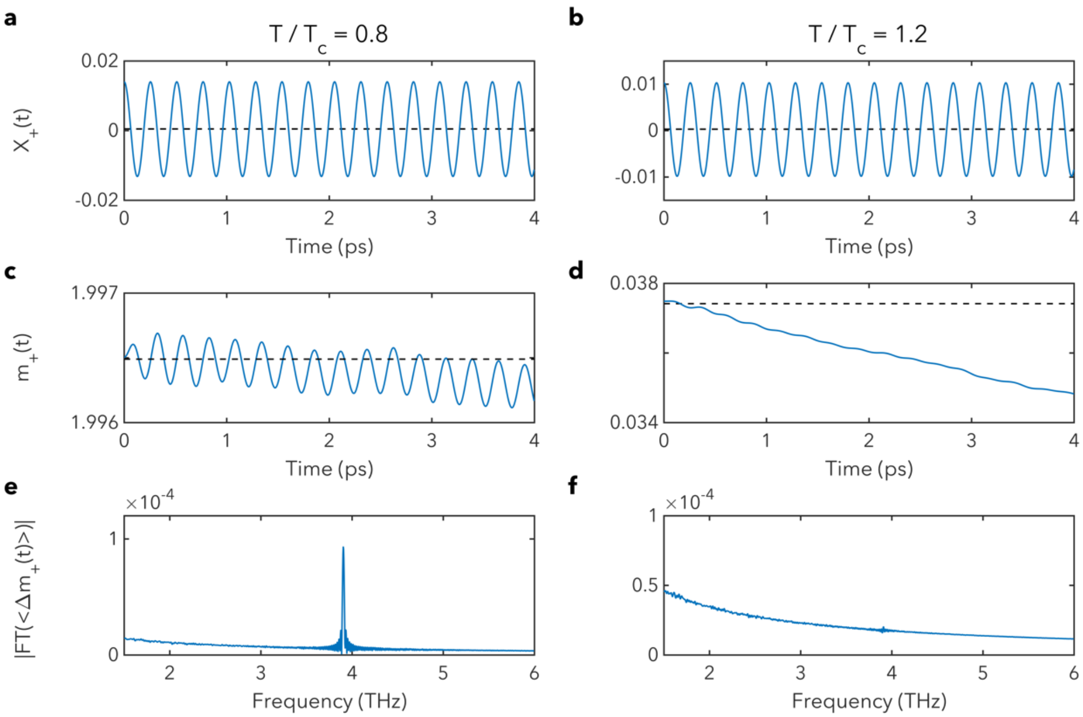

Supplementary Figure 9. Simulations of the vibrational and magnetization dynamics. Simulation results showing phonon coordinate coherent oscillations in both **a** the magnetically ordered phase at $T < T_c$ and **b** the paramagnetic regime for $T > T_c$. The dashed lines represent the thermodynamic equilibrium value. The magnetization shows coherent oscillations **c** in the FM phase below T_c, but not **d** in the paramagnetic phase above T_c. The Fourier transformation of the magnetization shows a pronounced peak at the phonon frequency **e** in the FM phase below T_c, but not **f** in the paramagnetic phase above T_c. All data shown assumes σ_+ polarized light.

Pump-Induced Dynamics: Coherent Oscillations

Here, we show two examples of datasets for the time evolution of the coupled spin-phonon model Hamiltonian. After application of the initial shift in lattice displacement, the phonon oscillates coherently, regardless of whether the system is in the magnetically ordered

phase or in the paramagnetic phase; see Supplementary Fig. 9a and Supplementary Fig. 9b, respectively. Qualitatively different behavior, however, is observed in the time dependence of the magnetization. While in the FM phase the coherent phonon oscillations effectively couple to the magnetization, yielding coherent magnetization oscillations (Supplementary Fig. 9c), such coupling is not observed above T_c. In the latter case the magnetization merely shows a demagnetizing drift, as expected after heating the system; short-time oscillations at the phonon frequency are not observed (Supplementary Fig. 9d) (note that the finite magnetization in the paramagnetic phase is an artifact of the finite system size, as also seen in Supplementary Fig. 6b). Consequently, a sharp phonon-induced signal in the Fourier spectrum of the magnetization is only visible below T_c (Supplementary Fig. 9e and Supplementary Fig. 9f).

Comparison of Different Models for the Spin-Phonon Dynamics

In the main manuscript we have employed a spin model based on the layered honeycomb model, in which the magnetic moments couple to local phonon modes at every lattice site. The model is chosen to closely resemble the bulk CrI_3 flakes that were used in the experiment. However, as we shall demonstrate below, the same phenomenology can also be observed for a simplified model of a single honeycomb layer, or even when the local phonon modes are replaced by a single global mode.

The full model is governed by the microscopic Hamiltonian $H = H_{\text{ph}} + H_{\text{sp}}$, with the phonon and spin contributions as specified in the main manuscript and parametrized by the spin exchange constants $(J_{H1}, J_{H2}, J_{H3}, J_K, J_D, J_A, J_{Hc})$. We refer to this model as the 3D model with local phonons. We now define a simplified single-layer model, i.e., we set the interlayer coupling $J_{Hc} = 0$ and refer to this as the 2D model with local phonons. When performing numerical

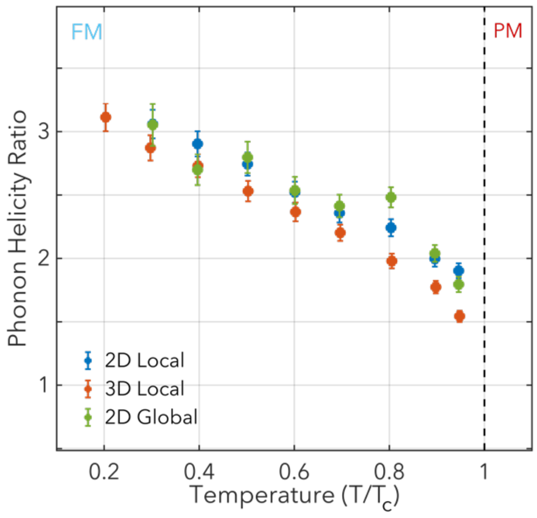

Supplementary Figure 10. Comparison of different models for spin-phonon dynamic simulations. The σ_+/σ_--ratio of integrated Fourier transform peaks at the A_{1g}^2 mode frequency is shown for three different models: a three-dimensional model for bulk CrI_3 with local phonon modes (red) and a two-dimensional model for single layer CrI_3 with either local phonon modes (blue) or global phonon modes (green).

simulations, it is thus sufficient to consider a single honeycomb layer of $2\times L\times L$ spins. From the 2D model with local phonons we further derive the 2D model with a single phonon mode by enforcing $X_\mathbf{r} = X_{\mathbf{r}'} \equiv X$ and $P_\mathbf{r} = P_{\mathbf{r}'} \equiv P$ for all lattice sites $\mathbf{r}$ and $\mathbf{r}'$, such that the model only has one effective global phonon mode. In the dynamic simulation, the pump pulse is then modeled as a change in the global phonon displacement X, which depends on the global average magnetization m, in analogy to the modeling that was used for local displacement $X_\mathbf{r}$ and local magnetization $m_\mathbf{r}$.

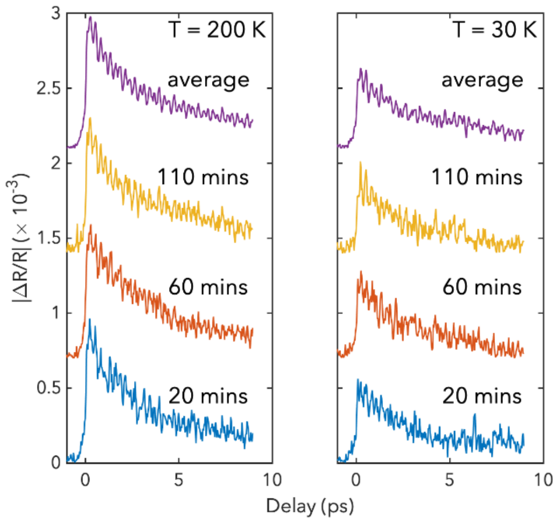

Supplementary Fig. 11. Long-time stability of the time-resolved differential reflectivity. Individual (i.e., not averaged) differential reflectivity traces taken at 20 minutes (blue), 60 minutes (red), and 110 minutes (yellow), as well as averages over 10 scans taken over the full 110 minutes measurement timeframe (purple) at 200 K and 30 K at a pump fluence of 0.64 mJ/cm^2.

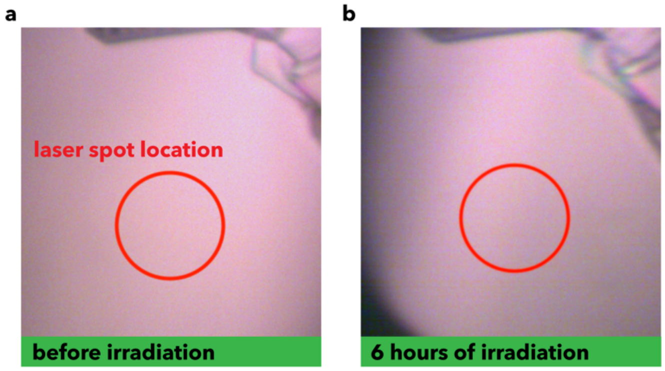

Supplementary Fig. 12. Sample quality before and after irradiation. Sample images taken using a 20X objective (a) before and (b) after 6 hours of continuous irradiation at 1.55 eV and a fluence of 0.64 mJ/cm^2. The location of the laser spot is denoted by the red circle.

The phonon helicity ratio plotted in Supplementary Fig. 10 shows no significant difference between the three different models. The small deviation that is discernible between the data for the 3D model and the data for the 2D models can be attributed to the slightly different onset of the magnetization curves for $T < T_c$.

Sample Quality Under Laser Irradiation

To confirm that the sample quality remained constant throughout the lifetime of the experiment at the 0.64 mJ/cm^2 pump laser fluence used in our experiments, we conducted time- resolved differential reflectivity measurements in a crossed polarized configuration above and below T_c. Supplementary Fig. 11 shows the results for the two cases. The left-hand panel shows individual (i.e., not averaged) time-resolved differential reflectivity scans taken at $T = 200$ K after 20 minutes (blue), 60 minutes (red), and 110 minutes (yellow) of continuous irradiation. No appreciable change in the traces is observed on these long timescales, and the signals are consistent with the average over 10 scans taken over the full 110 minutes experimental runtime shown in purple. We see no significant difference in the amplitude or decay rate of the coherent oscillations, nor in the overall exponential decay dynamics attributable to electron-phonon coupling. The same is true for the data taken at $T = 30$ K, as shown in the right-hand panel of Supplementary Fig. 11. Here, we see consistent results for scans taken at the three elapsed experimental times that are again mutually consistent with the 10-scan average. Finally, to further confirm that no sample degradation took place at our defined fluence, Supplementary Fig. 12 shows microscope images captured before and after 6 hours of continuous laser irradiation at 0.64 mJ/cm^2. Here the red circles indicate the location of the pump and probe focal spots, revealing no discernible darkening or other marring of the surface; these results clearly

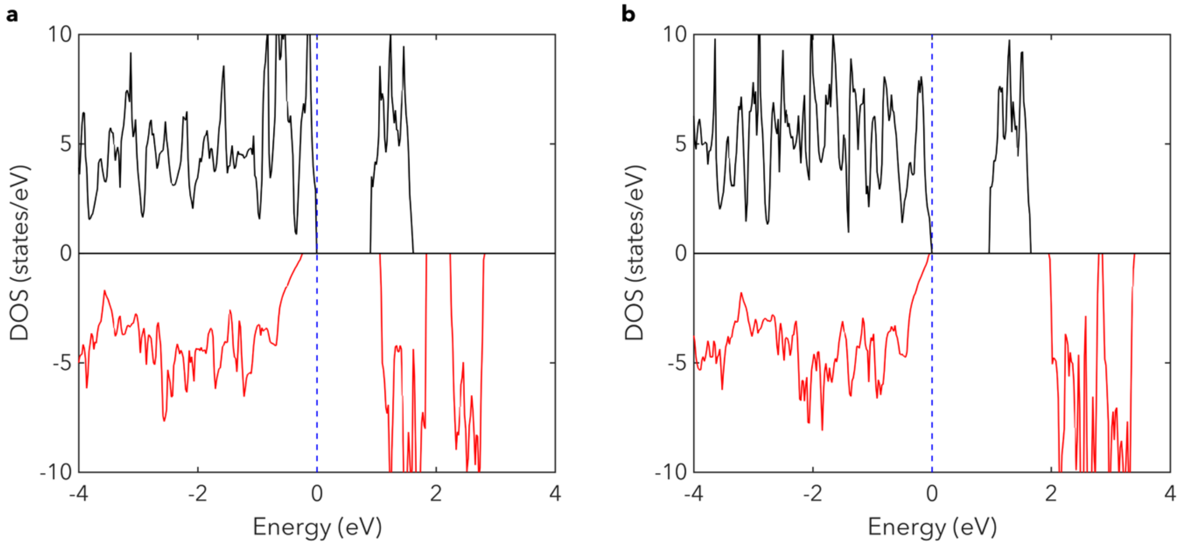

Supplementary Figure 13. Density of states for bulk CrI_3. Density of states (DOS) for bulk CrI_3 calculated with **a** LDA and **b** LDA+U. The spin-up component (black) has positive values, and the spin-down component (red) has negative values. The value of the Hubbard interaction on the Cr-3d orbitals is $U = 2.2$ eV.

demonstrate that the sample properties did not significantly change over the data acquisition time.

References

1. Wu, M., Li, Z., Cao, T. & Louie, S. G. Physical origin of giant excitonic and magneto-optical responses in two-dimensional ferromagnetic insulators. *Nat. Commun.* **10**, 2371 (2019).

2. Seyler, K. L. Probing valley and magnetic photoexcitations in 2D crystals and their heterostructures. (University of Washington, 2018).

3. Ščajev, P., Gudelis, V., Jarašiūnas, K. & Klein, P. B. Fast and slow carrier recombination transients in highly excited 4H– and 3C–SiC crystals at room temperature. *J. Appl. Phys.* **108**, 023705 (2010).

4. Carva, K., Battiato, M. & Oppeneer, P. M. Ab initio investigation of the Elliott-Yafet electron-phonon mechanism in laser-induced ultrafast demagnetization. *Phys. Rev. Lett.* **107**, 207201 (2011).

5. Park, J., Zhou, J.-J. & Bernardi, M. Spin-phonon relaxation times in centrosymmetric materials from first principles. *Phys. Rev. B* **101**, 045202 (2020).

6. Koopmans, B. *et al.* Explaining the paradoxical diversity of ultrafast laser-induced demagnetization. *Nat. Mater.* **9**, 259–265 (2010).

7. Sheng, H., Zhu, Y., Bai, D., Wu, X. & Wang, J. Thermoelectric properties of two-dimensional magnet CrI_3. *Nanotechnology* **31**, 315713 (2020).

8. McGuire, M. A., Dixit, H., Cooper, V. R. & Sales, B. C. Coupling of crystal structure and magnetism in the layered, ferromagnetic insulator CrI_3. *Chem. Mater.* **27**, 612–620 (2015).

9. Perry, D. L. *Handbook of Inorganic Compounds*. (CRC Press, 2011).

10. Lin, G. T. *et al.* Critical behavior of two-dimensional intrinsically ferromagnetic semiconductor CrI_3. *Appl. Phys. Lett.* **112**, 072405 (2018).

11. Liu, Y. & Petrovic, C. Three-dimensional magnetic critical behavior in CrI_3. *Phys. Rev. B* **97**, 014420 (2018).

12. Byrd, R. H., Gilbert, J. C. & Nocedal, J. A trust region method based on interior point techniques for nonlinear programming. *Math. Program.* **89**, 149–185 (2000).

13. McCreary, A. *et al.* Distinct magneto-Raman signatures of spin-flip phase transitions in CrI_3. *Nat. Commun.* **11**, 3879 (2020).

14. Jin, W. *et al.* Tunable layered-magnetism–assisted magneto-Raman effect in a two-dimensional magnet CrI_3. *Proc. Natl. Acad. Sci.* **117**, 24664–24669 (2020).

15. Meseguer-Sánchez, J. *et al.* Coexistence of structural and magnetic phases in van der Waals magnet CrI_3. *Nat. Commun.* **12**, 6265 (2021).

16. Chen, L. *et al.* Topological spin excitations in honeycomb ferromagnet CrI_3. *Phys. Rev. X* **8**, 041028 (2018).

17. Lee, I. *et al.* Fundamental spin interactions underlying the magnetic anisotropy in the Kitaev ferromagnet CrI_3. *Phys. Rev. Lett.* **124**, 17201 (2020).

18. Chen, L. *et al.* Magnetic anisotropy in ferromagnetic CrI_3. *Phys. Rev. B* **101**, 134418 (2020).

19. Xu, C., Feng, J., Xiang, H. & Bellaiche, L. Interplay between Kitaev interaction and single ion anisotropy in ferromagnetic CrI_3 and $CrGeTe_3$ monolayers. *npj Comput. Mater.* **4**, 57 (2018).

20. Stavropoulos, P. P. & Kee, H.-Y. Magnetic anisotropy in spin-3/2 with heavy ligand in honeycomb Mott insulators: application to CrI_3. *arXiv:2009.04475* 1–12 (2020).

21. Wahab, D. A. *et al.* Quantum Rescaling, Domain Metastability, and Hybrid Domain-Walls in 2D CrI_3 Magnets. *Adv. Mater.* **33**, 2004138 (2021).

22. Liu, L., Lin, Z., Hu, J. & Zhang, X. Full quantum search for high T_c two-dimensional van der Waals ferromagnetic semiconductors. *Nanoscale* **13**, 8137–8145 (2021).

23. Sandvik, A. W. Critical Temperature and the Transition from Quantum to Classical Order Parameter Fluctuations in the Three-Dimensional Heisenberg Antiferromagnet. *Phys. Rev. Lett.* **80**, 5196–5199 (1998).

24. Peczak, P., Ferrenberg, A. M. & Landau, D. P. High-accuracy Monte Carlo study of the three-dimensional classical Heisenberg ferromagnet. *Phys. Rev. B* **43**, 6087–6093 (1991).
